# A novel fast-slow model of diabetes progression: Insights into mechanisms of response to the interventions in the Diabetes Prevention Program

**DOI:** 10.1371/journal.pone.0222833

**Published:** 2019-10-10

**Authors:** Andrea De Gaetano, Thomas Andrew Hardy

**Affiliations:** 1 CNR-IASI BioMatLab (Italian National Research Council - Institute of Analysis, Systems and Computer Science - Biomathematics Laboratory), Rome, Italy; 2 Lilly Research Laboratories, Eli Lilly and Company, Lilly Corporate Center, Indianapolis, Indiana, United States of America; Indiana University Richard M Fairbanks School of Public Health, UNITED STATES

## Abstract

Several models for the long-term development of T2DM already exist, focusing on the dynamics of the interaction between glycemia, insulinemia and *β*-cell mass. Current models consider representative (fasting or daily average) glycemia and insulinemia as characterizing the compensation state of the subject at some instant in slow time. This implies that only these representative levels can be followed through time and that the role of fast glycemic oscillations is neglected. An improved model (DPM15) for the long-term progression of T2DM is proposed, introducing separate peripheral and hepatic (liver and kidney) insulin actions. The DPM15 model no longer uses near-equilibrium approximation to separate fast and slow time scales, but rather describes, at each step in slow time, a complete day in the life of the virtual subject in fast time. The model can thus represent both fasting and postprandial glycemic levels and describe the effect of interventions acting on insulin-enhanced tissue glucose disposal or on insulin-inhibited hepatic glucose output, as well as on insulin secretion and *β*-cell replicating ability. The model can simulate long-term variations of commonly used clinical indices (HOMA-B, HOMA-IR, insulinogenic index) as well as of Oral Glucose Tolerance or Euglycemic Hyperinsulinemic Clamp test results. The model has been calibrated against observational data from the Diabetes Prevention Program study: it shows good adaptation to observations as a function of very plausible values of the parameters describing the effect of such interventions as Placebo, Intensive LifeStyle and Metformin administration.

## Introduction

Mathematical modelling is being increasingly used in diabetology, in order to help explain the mechanisms of normal and diseased control of the glucose-insulin system, both in short-term dynamical perturbation experiments and in the long-term development of the disease [1–6]. In particular, in order to understand quantitatively the interplay between insulin sensitivity, pancreatic *β*-cell responsiveness and *β*-cell population dynamics, several mathematical models of the long-term development of Type 2 Diabetes Mellitus (T2DM) have been formulated [7–14]. Besides the Topp [7] and deWinter [9] models, which were extensively commented upon in comparison with our previous model [10], in the past few years contributions on long-term diabetes progression modelling by Bagust [8], Ribbing [11], Boutayeb [12], Palmer [13] and Ha [14] have appeared in the literature.

Bagust *et al*. [8] developed a first-principles spreadsheet model linking several clinically observable physiologic variables, essentially centered on HOMA. No equations or computational formulas are reported in this publication, and the model cannot therefore be reproduced in any way nor can the assumptions be quantitatively tested. However, these Authors show plausible curves of evolution of several clinical indices over long times, specifically highlighting the eventual therapeutic failure of the sequence of progressively more intensive treatment regimens they simulate (sulfonilureas, metformin, insulin etc.).

Ribbing *et al*. [11] used the original Topp [7] model to represent the relationship between fasting glycemia, fasting insulinemia and *β*-cell mass, as these evolve over the years. In order to adapt model forecasts to patient subgroups from the GLAD and GALLANT clinical studies, these Authors introduce at some point in the life of each subject a discrete step-up (OFFSET) in the fasting glycemia *“set-point”* for *β*-cell mass dynamics. Following this regime shift the patients, over time, become diabetic. In fact, these Authors state that “…Reduced insulin sensitivity alone does not cause diabetes…”.

Boutayeb *et al*. [12] introduce yet again another modification to the Topp [7] model by adding an *ϵ* factor (taking values between 0 and 1) multiplying the glucose toxicity term in Topp’s model, arguing therefore that with *ϵ* = 1 their *β*-cell dynamics equation reduces to that of Topp, while with *ϵ* = 0 no genetic predisposition to diabetes exists, no glucose toxicity occurs and no diabetes develops. They also replace the mass balance equations for glucose and insulin with more general equations involving Michaelis-Menten terms, without however clarifying the physiological basis of the new formulation. They finally conduct a local stability study of the qualitative behavior of the solutions of the model thus defined.

Palmer *et al*. [13] used our previously published model [10] as a basis for focusing on the effect of IL-1*β* (Inter-Leukin -1 *β*) inhibitors such as anakinra [15, 16]. They derived their parameter values from available literature sources and came to the conclusion that most of the effect of IL-1*β* blockers is likely due to improvement of insulin secretion by existing *β*-cells, whereas appreciable changes in *β*-cell mass could take several years to occur. This is consistent with the interpretation of the findings by Sloan-Lancaster *et al*. [17].

Ha *et al*. [14] again started from Topp’s model[7] adding to it a dose-response shift in glucose-stimulated insulin secretion (governed by a dynamically varying coefficient *γ*), and an increase in maximal insulin secretory capacity under persistent hyperglycemia (governed by a dynamically varying coefficient *σ*). In this way they introduced an intermediate time-scale between fast glucose-insulin equilibration and slow diabetes evolution. They calibrated parameter values for their model in order to represent the time-course of the disease in experimental ZDF (Zucker Diabetic Fatty) rats. These Authors reached the conclusion that an emergent threshold in glycemia separates two stability basins, one (at lower glycemia) leading to compensation, the other (at higher glycemia) leading to manifest diabetes. There are clear similarities between this work and our previous model [10], in that both predict the eventual fast acceleration of the development of diabetes once insulin hypersecretion slows down, and both account for the bistability of achievable compensation (in case of normoglycemia and/or maintained pancreatic reserve) or development of disease (in case of prolonged hyperglycemia with reserve exhaustion due to glucotoxicity).

We previously introduced a mathematical model of T2DM [10], which was able to replicate acceptably well the observed time courses of fasting glycemia and diabetes incidence [18], when compared quantitatively with the results of the non-intervention group in a large study of individuals at-risk for T2DM [the Diabetes Prevention Program (DPP) study [19–21]]. In addition to exploring physiological processes and the natural history of T2DM, such models can be utilized to predict the long-term effects of pharmacological or non-pharmacological interventions. To this end, our model was also able to replicate the effects of metformin, troglitazone, and intensive LifeStyle modification arms in the DPP study [18].

One limiting feature of our previous model, as well as other models which have appeared in the literature [7–14], was that the considered model structure did not allow for an independent assessment of post-prandial or post-OGTT (Oral Glucose Tolerance Test) glycemia, and lacked the ability to represent separately hepatic and peripheral insulin sensitivity. It was felt that this limitation was of practical importance in the use of the model for physiologically-based clinical trial simulation in diabetes and the exploration of potential effects of diverse pharmacologic mechanisms. We have therefore developed a new version of the Diabetes Progression Model (DPM). In this context, the mathematical approach used to reconcile daily glucose homeostasis (fast) with compensatory evolution of *β*-cell population and pancreatic reserve (slow) in the previous version of DPM (i.e. the consideration that in slow time fast variables are essentially at equilibrium, while in fast time slow variables are essentially constant) proved to be insufficient in capturing the possible effects of variable daily glycemia on long-term compensation mechanisms. Also, in the course of the study leading to the final form chosen for the new version of the model (DPM15), collateral issues arose, such as the representation of renal glucose reabsorption, or the possible modeling of Type 1 Diabetes Mellitus (T1DM) and its therapy. All of the above considerations determined a substantial restructuring of the whole modeling approach, which had the additional benefit of making the model versatile, allowing us to simulate with it other experimental conditions and perturbations (e.g. OGTT and Euglycemic Clamp).

The goal of the present work is to detail the assumptions underlying the functional form of the new model, named DPM15; to justify the numerical values assigned to its parameters; and to show model forecasts corresponding to all of the endpoints that were recorded in the DPP study (fasting glycemia and insulinemia, 30-min. glycemia and insulinemia during OGTT, 2-hr glycemia during OGTT).

## Materials and methods

The model to be described is named DPM15 since it is the 15th version of Diabetes Progression Model we built in the ongoing effort to capture the relevant features of the development of this disease. In the following, the model equations defining the model variables will be introduced and discussed. The model variables and parameters are summarized in Tables 1 and 2 respectively.

**Table 1:** Variables.

Variable	Units	Meaning
*B*	[*Mc*]	*β*-cell population size in Millions *β*-cells
*k*_*BB*_	[/*mo*]	*β*-cell net replication rate in fraction *β*-cells per month
*μ*	[/*mo*]	rate constant for additional *β*-cell mortality
*η*	[/*mo*]	*β*-cell replication reserve
*k*_*XηG*_	[/*mo*]	glucotoxicity (glucose-dependent pancreatic replication reserve decay) as modified by therapy
*k*_*η*_	[/*mo*^2^]	spontaneous recovery rate of the pancreas
*G*_*f*_	[*mM*]	fasting glycemia
A	[%]	glycosylated haemoglobin (percent)
*I*_*f*_	[*pM*]	fasting serum insulin concentration
*k*_*XI*_	[/*min*]	apparent first-order elimination rate constant for insulin
$k_{XGI}^{max}$	[/*min*]	maximal insulin-dependent tissue glucose uptake rate as modified by therapy
λ_*GI*_	[/*pM*/*mM*]	hepatic insulin sensitivity (natural value of insulin- and glucose-dependent HGO suppression) as modified by therapy
$k_{IB}^{max}$	[*pmol*/*min*/*Mc*]	maximal insulin secretion per Million *β*-cells as modified by therapy
*k*_*JS*_	[/*min*]	apparent first-order stomach emptying as modified by therapy
*G*_*f*24_	[*mM*]	FPG early next day
*G*_*η*_	[#]	weighted glycemia toxicity determining *η* suppression, as fraction of normal
*G*_*B*_	[*mM*]	weighted glycemia average stimulating *β*-cell replication
HomaIR	[(*μIU*/*ml*)*mM*]	homeostasis model assessment index of insulin resistance
HomaB	[(*μIU*/*ml*)/*mM*]	homeostasis model assessment index of *β*-cell function
Igenicx	[(*μIU*/*ml*)/*mM*]	Insulinogenic index
ClampM1	[*mg*/*kgBW*/*min*]	Clamp M value first step
ClampM2	[*mg*/*kgBW*/*min*]	Clamp M value second step
*S*	[*mmol*]	glucose content in the stomach
*J*	[*mmol*]	glucose content in the absorptive bowel (jejunum, ileum)
ra	[*mmol*/*min*]	rate of glucose appearance in the systemic circulation (from the gut)
*G*	[*mM*]	plasma glucose concentration (in fast time)
*L*	[*pM*]	serum glucagon concentration (in fast time)
*I*	[*pM*]	serum insulin concentration (in fast time)
*q*	[*mmol*/*z*]	density of glucose amount in tubule with respect to normalized tubule length
*v*	[*L*/*z*]	density of tubular water volume with respect to normalized tubule length
*C*	[*mM*]	concentration of glucose in pre-urine
ur	[*mmol*/*min*]	rate of urinary glucose loss

**Table 2:** Parameters.

Parameter	Units	Meaning	Value
*t*_0_	[*mo*]	starting age for numerical integration of slow model, in months	0
*t*_*end*_	[*mo*]	final age for numerical integration of slow model, in months	1080
*B*_*max*_	[*Mc*]	maximal *beta*-cell population size (carrying capacity)	4000
*B*_0_	[*Mc*]	baseline value of *B* at slow initial time (age *t*_0_)	1000
*ν*_*BG*_	[#]	exponent of the Hill function describing replication stimulation by glycemia	2
*G*_*B*50_	[*mM*]	glycemia of half-maximal *beta*-cell replication stimulation	9
*η*_0_	[/*mo*]	baseline value of *η* at slow initial time (age *t*_0_)	0.04
*k*_*XηG*0_	[/*mo*]	baseline value of *k*_*XηG*_ at slow initial time (age *t*_0_)	0.02
*k*_*ηendprop*_	[#]	Level of *k*_*η*_ at the end of life (at *t*_*end*_) as proportion of *k*_*η start*_	0.4
*k*_*GG*_	[/*mo*]	rate of convergence of fasting glycemia from start-of-day to end-of-day values	0.4
*G*_*f*0_	[*mM*]	fasting glycemia at age t0	4.2
*k*_*XA*_	[/*mo*]	spontaneous elimination rate constant of (glycosylated) Haemoglobin	0.4
*A*_0_	[%]	baseline value of *A* at slow initial time (age *t*_0_)	5
*V*_*G*_	[*L*/*kg*]	glucose distribution volume	0.19
*V*_*I*_	[*L*/*kg*]	insulin distribution volume	0.19
*W*	[*kg*]	experimental subject’s body weight	70
*k*_*XIstart*_	[/*min*]	apparent first-order elimination rate constant for insulin at baseline (at age *t*_0_)	0.05
*k*_*XIend*_	[/*min*]	apparent first-order elimination rate constant for insulin at the end of a normal life (e.g. at age 90 years)	0.045
$k_{XGI0}^{max}$	[/*min*]	baseline value of $k_{XGI}^{max}$ at slow initial time (age *t*_0_)	0.08
$k_{XGI0}^{0}$	[/*min*/*pM*]	baseline value of $k_{XGI}^{0}$ at slow initial time (age *t*_0_)	0.00015
$f_{kXGI}^{min}$	[#]	minimum value possible for $k_{XGI}^{max}$ as proportion of its baseline value at age *t*_0_	0.05
*t*_*kXGIstart*_	[*mo*]	starting time of $k_{XGI}^{max}$ decrease	216
*ν*_*kXGI*_	[#]	exponent for $k_{XGI}^{max}$ decrease	3
*t*_*kXGI*50_	[*mo*]	time of half-maximal $k_{XGI}^{max}$ decrease	800
λ_*GI*0_	[/*pM*/*mM*]	baseline value of λ_*GI*_ at slow initial time (age *t*_0_)	0.015
$f_{\lambda GI}^{min}$	[#]	minimum value possible for λ_*GI*_ as proportion of its baseline value at age *t*_0_	0.05
*t*_λ*GIstart*_	[*mo*]	starting time of λ_*GI*_ decrease	216
*ν*_λ*GI*_	[#]	exponent for λ_*GI*_ decrease	8
*t*_λ*GI*50_	[*mo*]	time of half-maximal λ_*GI*_ decrease	550
$k_{IB0}^{max}$	[*pmol*/*min* /*Mc*]	baseline value of $k_{IB}^{max}$ at slow initial time (age *t*_0_)	0.5
$f_{kIB}^{min}$	[#]	minimum value possible for $k_{IB}^{max}$ as proportion of its baseline value at age *t*_0_	0.25
*t*_*kIBstart*_	[*mo*]	starting time of $k_{IB}^{max}$ decrease	216
*ν*_*kIB*_	[#]	exponent for $k_{IB}^{max}$ decrease	2.5
*t*_*kIB*50_	[*mo*]	time of half-maximal $k_{IB}^{max}$ decrease	950
*k*_*JS*0_	[/*min*]	baseline value of *k*_*JS*_ at slow initial time (age *t*_0_)	0.0235
*α*_*LyKxgi*_	[/*mo*]	rate of onset of effect of Ly on *k*_*XGI*_	0.1
*β*_*LyKxgi*_	[/*mo*]	rate of decay of effect of Ly on *k*_*XGI*_	0.009
LyKxgiCurr	[#]	maximal effect of Ly therapy as proportional increase of *k*_*XGI*_ above current level.	0
*α*_*LyLamgi*_	[/*mo*]	rate of onset of effect of Ly on λ_*GI*_	0.3
*β*_*LyLamgi*_	[/*mo*]	rate of decay of effect of Ly on λ_*GI*_	0.01
LyLamgiCurr	[#]	maximal effect of Ly therapy as proportional increase of λ_*GI*_ above current level.	0
*α*_*LyKjs*_	[/*mo*]	rate of onset of effect of Ly on *k*_*JS*_	0.05
*β*_*LyKjs*_	[/*mo*]	rate of decay of effect of Ly on *k*_*JS*_	0.002
LyKJS	[#]	Effect of Ly therapy on gastric emptying rate *k*_*JS*_ as fraction of current value, when positive accelerates gastric emptying	0
*τ*_0_	[*min*]	starting time for numerical integration of fast model, in minutes after midnight	360
*τ*_*end*_	[*min*]	final time for numerical integration of fast model, in minutes	1800
*τ*_1_	[*min*]	time of breakfast in minutes after midnight	420
$M_{1}^{\text{gluc}}$	[*mmol*]	breakfast contribution to circulating glucose	417
*τ*_2_	[*min*]	time of lunch in minutes after midnight	720
$M_{2}^{\text{gluc}}$	[*mmol*]	lunch contribution to circulating glucose	280
*τ*_3_	[*min*]	time of dinner in minutes after midnight	1080
$M_{3}^{\text{gluc}}$	[*mmol*]	dinner contribution to circulating glucose	280
$k_{Xg\;brox}^{max}$	[*mmol* /*kgBW* /*min*]	maximal brain glucose oxidation	0.0059
*G*_*brox*50_	[*mM*]	glycemia of half-maximal brain glucose oxidation	0.5
*k*_*GJ*_	[/*min*]	transfer rate constant from intestine to plasma, absorption rate	0.025
*f*_*GJ*_	[#]	proportion of absorbed nutrients entering the circulation	0.9
*k*_*XG*_	[/*min*]	first-order insulin-independent glucose tissue uptake rate	0.001
$k_{GI}^{max}$	[*mmol* /*min*]	maximal rate of insulin-dependent hepatic glucose production	0.75
*L*_*G*50_	[*pM*]	glucagon concentration of half-maximal stimulation of gluconeogenesis or glycogen lysis	0.05
*L*_0_	[*pM*]	fasting Glucagon plasma concentration	15
*k*_*XL*_	[/*min*]	first-order glucagon elimination rate from plasma	0.04
$f_{LG}^{min}$	[#]	minimum possible value for glucagon secretion rate as proportion of its max value at zero glycemia	0.15
λ_*LG*_	[/*mM*]	exponential rate of decay of glucagon secretion with increasing glycemia	0.55
*f*_*IJ*_	[#]	maximum jejunal glucose content additional effect (as proportion of plasma glucose effect) towards insulin secretion by pancreas, max incretin effect	1.15
*J*_*G*50_	[*mmol*]	jejunal glucose content at which incretin effect is half-maximal	200
*ν*_*IG*_	[#]	exponent for increase in *β*-cell-mass-specific insulin secretion rate with increasing glycemia	3
*G*_*I*50_	[*mM*]	glycemia of half-maximal *β*-cell-mass-specific insulin secretion rate	14.4
ClInulin	[*L*/*kgBW* /*min*]	Inulin clearance or glomerular filtration rate	0.0018
Furine	[*L*/*kgBW* /*min*]	Urinary flow	0.00002
*D*_*u*_	[/*z*^2^]	tubular glucose diffusion coefficient	0
$k_{GU}^{max}$	[*mmol* /*kgBW* /*min*]	maximal rate of glucose transfer from pre-urine to plasma (glucose reabsorption)	0.027
*C*_*GU*50_	[*mM*]	tubular glucose concentration of half-maximal transport	19
λ_*VZ*_	[/*z*]	Exponent for decay of water or volume flow along tubule	8
λ_*QZ*_	[/*z*]	Exponent for decay of glucose reabsorption along tubule	7
*I*_*f*0_	[*pM*]	fasting insulinemia at age *t*_0_	19.8
*I*_*KXGI*50_	[*pM*]	insulinemia of haf-maximal effect on peripheral tissue glucose uptake	333
$k_{GL}^{max}$	[*mmol* /*min*]	maximal rate of glucagon-dependent, treatment-insensitive hepatic glucose production	0.0685
$k_{LG}^{max}$	[*pM*/*min*]	maximal rate of glucose-dependent glucagon secretion effect on glucagon concentration	2.56
*k*_*η start*_	[/*mo*^2^]	spontaneous recovery rate of the pancreas at *t*_0_	0.00085
$k_{BB}^{min}$	[/*mo*]	minimum value of the replication rate *k*_*BB*_	-0.0104
*k*_*AG*_	[%/*mo* /*mM*]	rate constant of production of glycosylated haemoglobin from circulating glucose	0.395

In the Parameters table (Table 2) the Value reported refers to the calibrated “baseline” value, the hypothetical value in the untreated DPP cohort. All DPP treatment arms shared these same general parameter values except for those specific parameters (Table 3) embodying the differences between groups induced by the different treatments.

**Table 3:** Parameter value configurations.

Parameter	Units	DPP no treatment	DPP Placebo	DPP LifeStyle	DPP Metformin
*α*_*LyKxgi*_	[/*mo*]	0.1	0.03	0.1	0.03
*β*_*LyKxgi*_	[/*mo*]	0.009	0.012	0.009	0.012
LyKxgiCurr	[#]	0	2.3	4.2	2.3
*α*_*LyLamgi*_	[/*mo*]	0.3	0.3	0.5	0.3
*β*_*LyLamgi*_	[/*mo*]	0.01	0.025	0.03	0.022
LyLamgiCurr	[#]	0	0.2	1.2	1.2
LyKJS	[#]	0	0.05	-0.05	0.1

Aspects of the model that are retained from the previous model are discussed here only briefly. Further details can be found in DeGaetano *et al*. [10]. This holds in particular for the choice of parameter values as distilled from the literature or from the adaptation of specific sub-models to available observations.

In the present work, a rather mechanistic, physiologic approach has been followed, explicitly computing the daily time-course of glycemia, insulinemia and related variables, at each step of the numerical integration of the slow system (say, every month), using a model of fast glucose homeostasis. From this daily portrait, the desired target descriptors of glucose/insulin control at that moment in slow time may be computed (e.g. average daily insulinemia or glycemic Area Under the Curve AUC after meals), and their value used to affect the further evolution of the slow pancreatic compensation system. In this way, a kind of alternating-step solution is obtained: the overall compensation status as reflected by the current values of the slow model variables (e.g. *β*-cell mass) is used as framework for the reconstruction of the daily profiles of fast model variables. In turn, fast variables can impact slow variables (e.g. potential effects of daily glycemia on *β*-cell replication) and these effects are allowed to integrate over another slow time interval. The model structure described above portrays explicitly the interplay of a slow model for the evolution of pancreatic compensation and changes in insulin sensitivity, with a fast model for immediate glucose/insulin homeostasis. The possibility of availing oneself of a (simplified but) complete description of glucose homeostasis at any time in the life of the subject allows the investigator to study new issues, which a solely slow model could not address: such are, for instance, the implications of assuming *β*-cell glucose toxicity to be due to elevated peak rather than average glycemias. Another new issue that can be addressed by this change of strategy is that of explicitly modeling, at any given time in the patients life, some clinical indices (e.g. HOMA-IR and HOMA-B) and the expected response of the subject to some commonly used experimental perturbation procedures, such as the Intra-Venous Glucose Tolerance Test (IVGTT), the OGTT or the Euglycemic Hyperinsulinemic Clamp (EHC). In this way the investigator has the possibility of correlating directly the hypothesized lifetime evolution with observed experimental measures and commonly employed clinical indices.

Given the slow-fast structure of the model, in order to avoid confusion when referring to “time”, the letter t (months) has been reserved for slow time, indicating the evolution of overall compensation on a scale of months to years, while the letter *τ* (minutes) has been used to indicate the evolution of the glucose homeostasis mechanism after acute perturbations such as meals.

### Slow model

#### *β*-cell mass (B)

Eq 1 defines the variation of the *β*-cell population *B* as depending on a (variable) net replication coefficient *k*_*BB*_ and on a possible additional coefficient of *β*-cell mortality *μ*, through which it is possible, for instance, to represent the early auto-immune development of T1DM or the postulated cytotoxic effects of cytokines or lipid species in T2DM. Besides the introduction of *μ*, the form of the equation differs from the previous model in that a limiting ‘carrying capacity’ for B has been introduced, transforming the previous exponential model [10] into the present logistic model. The value of the carrying capacity *B*_*max*_ has been set at 4 billion cells, i.e. four times the normal value of approximately 1 billion *β*-cells previously estimated on the basis of several literature sources [22–27] and assumptions about cell size. Pregnancy and obesity may be associated with a doubling of *β*-cell mass [28, 29]. A maximal four-fold increase does not seem unreasonable and may allow ample space for normal variation.

$$\begin{array}{c} \frac{dB(t)}{dt}=\;\;k_{BB}(\eta ,\;G_{B})\;\;\;B\;\;\;(1\;\;-\;\;\frac{B}{B_{\max}})\;\;-\;\;\mu \;B\;\;\;,\;\;\;\;\;\;B(t_{0})\;\;=\;\;B_{0} \end{array}$$ (1)

#### *β*-cell net replication rate (*k*_*BB*_)

A fundamental assumption of the present model is the dual effect of glycemia on *β*-cell replication: *β*-cell population dynamics is affected by hyperglycemia through both a direct short-term stimulation [30–32] and an indirect longer-term inhibition of net replication, possibly due to glucose toxicity “exhausting” *β*-cell replication reserve [22, 33–36]. The same assumption underlies our previous model [10] and is similar to what was postulated by Topp *et al*. [7].

Eq 2 relates the replication coefficient *k*_*BB*_ (*β*-cell net replication rate) with (short-term) glycemic stimulation:

$$\begin{array}{c} k_{BB}(\eta ,G_{B})\;\;\;=\;\;\;k_{BB}^{\min}\;\;+\;\;\eta \;\;\frac{G_{B}^{\nu_{BG}}}{G_{B50}^{\nu_{BG}}+G_{B}^{\nu_{BG}}}\;\;,\;\;\;\;\;k_{BB}(t_{0})\;\;=\;k_{BB}(\eta_{0},G_{B0})\;\;=\;\;0 \end{array}$$ (2)

The net replication rate is increased by hyperglycemia (with a nonlinear, saturating mechanism depending on pancreatic replication reserve *η*) above a minimum rate $k_{BB}^{min}$, taken to be negative. In this way, allowance is made for both positive and negative oscillations of *β*-cell net replication rate, translating into increments and decrements of *β*-cell mass. Notice that in this formulation *G*_*B*_ is some function assumed to best describe the aggregated effect of the daily glycemic variations in stimulating *β*-cell replication: for the current implementation of the model, *G*_*B*_ has been taken simply as average daily glycemia.

#### *β*-cell replication reserve (*η*)

The possible excursion of the *β*-cell net replication rate *k*_*BB*_ has been termed $\eta =k_{BB}^{max}-k_{BB}^{min}$. It is non-negative and is governed by Eq 3:

$$\begin{array}{c} \frac{d\;\eta (t)}{dt}=\;\;\;-k_{X\eta G}\;\;G_{\eta }\;\;\eta \;\;\;+\;\;k_{\eta },\;\;\;\;\;\;\eta (t_{0})\;\;=\;\;\eta_{0} \end{array}$$ (3)

This excursion or range is a measure of the maximal replication rate of the pancreatic *β*-cells as depending on the current state of pancreatic “health”: in other words, it represents pancreatic *β*-cell replication reserve. It has some starting value *η*_0_ and then increases to a maximum *η*_*max*_ or decreases towards zero depending on glycemia levels. Hyperglycemia is supposed to be toxic to *β*-cell replication [33, 34], hence sustained hyperglycemia will lead to a decrease of *η*. Notice that setting *k*_*η*_ at zero, we would assert that pancreatic reserve necessarily decreases with age.

The function *G*_*η*_ is computed as the integrated mean over 24 hours (computed from the fast daily model) of the glucose toxicity produced by the varying glucose concentrations throughout the day. Glucose toxicity is monotonically increasing with glycemia, has been calibrated on TUNEL (Terminal deoxynucleotidyl transferase dUTP nick end labeling) data [37, 38] and indicates *η* suppression as a fraction of normal (normal = 1 = 100% at fixed 5.5 mM glycemia). In other words, at each instant in fast time the current glycemia determines the current glucose toxicity following Maedler *et al*. [37, 38] (according to an increasing, saturating Hill function with 0 toxicity at 0 mM glycemia, toxicity 1 at 5.5 mM, toxicity 3.5 at 30 mM and asymptotical toxicity 4 at infinite glycemia); toxicities throughout the day are integrated and then divided by the day’s duration in order to obtain the integrated average toxicity *G*_*η*_ for that day.

#### Pancreatic glucose toxicity

In Eq 3, *k*_*XηG*_ is the coefficient expressing the intensity of pancreatic glucose toxicity. It may be allowed to vary over time, starting at some value *k*_*XηG*0_ and being possibly modified by therapy.

#### Glycemia-independent *β*-cell mortality rate (*μ*)

Independent factors, such as inflammation or auto-immune processes, may contribute to *β*-cell mortality independently of glycemic levels. Eq 4 allows the expression of this excess mortality rate *μ* over time, starting from a level *μ*_0_ (assumed to be zero in health) and progressing sigmoidally towards a maximum additional mortality rate *μ*_max_:

$$\begin{array}{c} \mu (t)\;\;\;=\;\;\;\{\begin{array}{c} \mu_{0}\;+\;\;(\mu_{\max}\;\;-\;\;\mu_{0})\;\;\frac{{\left(t\;\;-\;\;t_{\mu \;start}\right)}^{\nu_{\mu }}}{{\left(t_{\mu \;50}\;\;-\;\;t_{\mu \;start}\right)}^{\nu_{\mu }}+\;\;{\left(t\;\;-\;\;t_{\mu \;start}\right)}^{\nu_{\mu }}}\;\;,\;\;\;\;\;t\;\;\geq \;\;t_{\mu \;start}\\ \mu_{0}\;\;\;\;otherwise \end{array} \end{array}$$ (4)

This equation is used, for instance, when using the model to represent the time course of Type 1 Diabetes Mellitus (T1DM), when additional *β*-cell mortality leads to rapid disappearance of most of the *β*-cell population over a relatively short time period.

#### Spontaneous pancreatic recovery rate

In Eq 5, *k*_*η*_ indicates the current ability of the pancreas to increase (recover) its *β*-cell proliferation rate. This ability is assumed to vary linearly between *t*_0_ and *t*_*end*_ (from young age to end of life) so as to allow the possibility of representing non-constant spontaneous pancreatic recovery rate throughout the subject’s lifetime. This is of interest when considering that natural aging may reduce, over time, *β*-cell proliferative capacity [39, 40].

$$\begin{array}{c} k_{\eta }(t)\;\;\;=\;\;\;k_{\eta start}\;\;(1\;\;+\;\;\;\frac{t-t_{0}}{t_{end}-t_{0}}(k_{\eta \;end\;prop}-1))\;\; \end{array}$$ (5)

#### Fasting plasma glucose concentration

Given the current slow state, a Daily run of the fast model, inclusive of meals etc., when starting at glycemia *G*_*f*_ calculates glycemia *G*_*f*24_ exactly 24 hours later. In the long run therefore, *G*_*f*_ will tend to the value *G*_*f*24_ over slow time, and this is assumed in the model to occur at a rate *k*_*GG*_. In general, we do not expect *G*_*f*_ to be equilibrated within a day, in particular when glycemia is in a rising phase (e.g. in the pre-diabetic state). Writing Eq 6 we recognize that slow *G*_*f*_(*t*) may differ from the corresponding slow *G*_*f*24_(*t*) and assume that *G*_*f*_ tends to *G*_*f*24_ (in the pre-diabetic state example, we assume it increases towards *G*_*f*24_) with rate *k*_*GG*_. Equilibrium may in fact never be attained, because as *G*_*f*_ converges to *G*_*f*24_, *G*_*f*24_ itself may shift due to the concurrent change in slow model variables. It is to be noticed here how the concept of the convergence of *G*_*f*_ to *G*_*f*24_ provides the link between fast time and slow time glycemia, so that changes in *G*_*f*24_ due to fast-time dynamics in fact drive the evolution of the whole system of glucose homeostasis in slow time.

$$\begin{array}{c} \frac{dG_{f}(t)}{dt}=\;\;k_{GG}(G_{f24}\;-\;G_{f})\;\;\;\;\;,\;\;\;\;\;\;G_{f}(t_{0})\;\;=\;\;G_{f0} \end{array}$$ (6)

#### Fasting serum insulin concentration

The fasting serum insulin concentration is computed as the fast-equilibrium value at current *β*-cell mass and fasting glycemia values. The function $\psi_{I}^{gluc}\left(B,G\right)$ indicates glucose-driven pancreatic insulin secretion at given *β*-cell mass *B* and driving glycemia *G*:

$$\begin{array}{c} I_{f}(t)\;\;\;=\;\;\;\;\;\frac{\psi_{I}^{gluc}\;(B(t),\;\;G_{f}(t))}{V_{I}\;W\;k_{XI}}\;\;,\;\;\;\;\;I_{f}(t_{0})\;\;\;=\;\;I_{f0} \end{array}$$ (7)

$$\begin{array}{c} \psi_{I}^{gluc}\;(B,G)\;\;\;=\;\;\;k_{IB}^{max}\;B\;\;\frac{G^{\nu_{IG}}}{G_{I50}^{\nu_{IG}}\;+\;G^{\nu_{IG}}} \end{array}$$ (8)

In Eq 8 insulin secretion depends therefore on current *β*-cell mass *B*, on $k_{IB}^{max}$, the glucose sensitivity of the existing *β*-cells, and on a saturating stimulus provided by increasing glucose concentrations.

#### Difference of exponentials

In the following, we use a standard difference-of-exponentials functional form to express the time-course of the action of a given therapy on some control variable, i.e. on some key state variable determining the evolution of the whole system:

$$\begin{array}{c} f_{exp}\left(Ampl,\alpha ,\beta ,t\right)=Ampl\frac{\left(e^{-\beta \;t}-e^{-\alpha \;t}\right)}{peak\left(\alpha ,\beta \right)},\;t\geq 0 \end{array}$$ (9)

$$\begin{array}{c} peak(\alpha ,\beta )\;\;\;=\;\;\;(e^{-\beta \;\;\;t_{peak}(\alpha ,\beta )}-e^{-\alpha \;t_{peak}(\alpha ,\beta )}) \end{array}$$ (10)

$$\begin{array}{c} t_{peak}(\alpha ,\beta )\;\;\;=\;\;\;\frac{\log(\alpha /\beta )}{\alpha -\beta }\; \end{array}$$ (11)

The difference of exponentials defines a curve starting from 0 at time 0, rising to a peak amplitude *Ampl* and redescending to zero (*α* > *β*) as *t* → ∞, with respective rates of increase and decrease defined by the relative values of *α* and *β*, the larger the *α* the faster the rise, the larger the *β* the faster the fall.

Using the difference of exponential formula, it is possible to specify a progressive rise of the effect of therapy on some control variable, starting at some therapy initiation time *t*_*thx*_.

Notice how, by changing the values of *α* and *β* appropriately, it is possible to specify very different time courses of the effect: for instance, with *α* very large and *β* very small, the difference of exponentials can approximate a step function, with immediate increase and essentially permanent effect.

#### Insulin secretory function

We indicate with ${\tilde{k}}_{IB}^{max}$ the “natural” maximal insulin production rate per million *β*-cells, which can be made to vary (decrease) over time in order to express a possible decay of functional secreting ability:

$$\begin{array}{c} {\tilde{k}}_{IB}^{max}\;\;\;=\;\;\;\{\begin{array}{c} \;\;\;k_{IB0}^{max}(1-(1-f_{kIB}^{min})\frac{{\left(t-t_{kIBstart}\right)}^{\nu_{kIB}}}{{\left(t_{kIB50}-t_{kIBstart}\right)}^{\nu_{kIB}}\;\;+\;\;{\left(t-t_{kIBstart}\right)}^{\nu_{kIB}}})\;\;\;\;\;\;\;\;\;,t>t_{kIBstart}\\ k_{IB0}^{max}\;\;\;\;\;\;\;\;\;\;\;\;\;\;\;\;\;\;\;\;\;\;\;\;\;\;\;\;\;\;\;\;\;\;\;\;\;\;\;\;\;\;\;\;\;\;\;\;\;\;\;\;\;\;\;\;\;\;\;,\;\;\;t\leq t_{kIBstart} \end{array} \end{array}$$ (12)

so that *t*_*kIB*_ is, for any value of *ν*_*kIB*_, the time at which ${\tilde{k}}_{IB}^{max}$ has changed by 50% of $k_{IB}^{max}(1-f_{kIB}^{min})$. In the present work, we assume insulin secretion ability to (possibly) decrease over time: by suitably varying the parameters, the model may represent insulin secretory function becoming severely impaired at different epochs in the subject’s lifetime (early, late or never, by increasing *t*_*kIB*50_), changing less or more suddenly (by increasing *ν*_*kIB*_), beginning to deteriorate earlier or later (by increasing *t*_*kIBstart*_), starting with a smaller or larger value ($k_{IB}^{max}$) and attaining eventually a smaller or larger proportion of the initial value ($f_{kIB}^{min}$). This “natural” value ${\tilde{k}}_{IB}^{max}$ may then be modified by therapy to yield the actual current value $k_{IB}^{max}$.

#### Insulin elimination rate

Over time, the apparent elimination rate of insulin from serum may be supposed to vary. In fact, it is presumed to be slowly decreasing over lifetime [41] (i.e. *k*_*XIend*_ < *k*_*XIstart*_):

$$\begin{array}{c} \;k_{XI}(t)\;\;\;=\;\;\;k_{XIstart}\;\;+\;\;\;\frac{t-t_{0}}{t_{end}-t_{0}}(k_{XIend}-k_{XIstart}) \end{array}$$ (13)

#### Peripheral insulin sensitivity

In order to represent variable maximal insulin-dependent tissue glucose uptake (Peripheral Glucose Disposition, PGD) rate, we may take the “natural” value of $k_{XGI}^{max}$ to be represented by:

$$\begin{array}{c} {\tilde{k}}_{XGI}^{max}\;\;\;=\;\;\;\{\begin{array}{c} \;\;\;k_{XGI0}^{max}(1-(1-f_{kXGI}^{min})\frac{{\left(t-t_{kXGIstart}\right)}^{\nu_{kXGI}}}{{\left(t_{kXGI50}-t_{kXGIstart}\right)}^{\nu_{kXGI}}\;\;+\;\;{\left(t-t_{kXGIstart}\right)}^{\nu_{kXGI}}})\;\;\;\;\;\;\;\;\;,t>t_{kXGIstart}\\ k_{XGI0}^{max}\;\;\;\;\;\;\;\;\;\;\;\;\;\;\;\;\;\;\;\;\;\;\;\;\;\;\;\;\;\;\;\;\;\;\;\;\;\;\;\;\;\;\;\;\;\;\;\;\;\;\;\;\;\;\;\;\;\;\;,\;\;\;t\leq t_{kXGIstart} \end{array} \end{array}$$ (14)

Expressing the possible decay of insulin sensitivity over time in the same form as that used above for the time-course of insulin secretory function offers a large flexibility in the kind of behavior that can be represented (early or late start, fast or slow decrement, large or small eventually preserved function etc.). The “natural” value of peripheral insulin sensitivity ${\tilde{k}}_{XGI}^{max}$, determined by a combination of genetic factors, lifestyle etc., is then possibly modified by therapy, with effect expressed by *f*_*exp*_(LyKxgiCurr, *α*_*LyKxgi*_, *β*_*LyKxgi*_, *t* − *t*_*thx*_) at any given time after the initiation of therapy, so as to yield the actual current value $k_{XGI}^{max}$.

We note that hepatic glucose uptake is here comprised in the total Peripheral Glucose Disposition, and is considered to be insulin sensitive in the normal individual. There has been debate about this point. It is true that the hepatic glucose transporter (GLUT2) is not responsive to insulin [42]. It is also true that some studies have not shown stimulation of hepatic glucose uptake by hyperinsulinemia. Nevertheless, compelling evidence exists for insulin stimulation of hepatic glucose uptake [43–45]. While the assumption, that the effect of insulin on hepatic (or splanchnic) glucose uptake is similar to insulin-dependent glucose disposition in other tissues, is clearly an oversimplification of the differential mechanisms of insulin stimulated glucose uptake in liver and peripheral tissues, the above literature supports similar effects and overlapping concentration-responses in liver and other insulin-responsive tissues. The assumption, that we may model with a single overall effect the composition of peripheral and hepatic glucose uptake, seems not unreasonable in the light of the above considerations.

#### Hepatic insulin sensitivity

In the present version of the model, *“Hepatic Insulin Sensitivity”* refers to the action of insulin to decrease hepatic glucose output (HGO) and also kidney glucose output to the extent that it is significant. More specifically, hepatic insulin sensitivity (λ_*GI*_) is formalized as the rate of the exponential decay of hepatic glucose output with increasing glycemia and insulinemia (see Eq 20). This relationship is supported by experimental observations [46, 47]. Note that this expression only reflects the effect of insulin on hepatic glucose production and not on hepatic glucose uptake (so that HGO is always non-negative in our model). Instead, the effect of insulin on hepatic (or splanchnic) glucose uptake is assumed to be similar to insulin-dependent glucose disposition in other tissues and is therefore incorporated in the *k*_*XGI*_ (peripheral insulin sensitivity) term. The study by Basu and colleagues [45] supports this assumption.

In order to represent variable hepatic insulin sensitivity (decreasing over slow time), we may take its “natural” value ${\tilde{\lambda }}_{GI}$ to be represented by:

$$\begin{array}{c} {\tilde{\lambda }}_{GI}\;\;\;=\;\;\;\{\begin{array}{c} \;\;\;\lambda_{GI0}(1-(1-f_{\lambda GI}^{min})\frac{{\left(t-t_{\lambda GIstart}\right)}^{\nu_{\lambda GI}}}{{\left(t_{\lambda GI50}-t_{\lambda GIstart}\right)}^{\nu_{\lambda GI}}\;\;+\;\;{\left(t-t_{\lambda GIstart}\right)}^{\nu_{\lambda GI}}})\;\;\;\;\;\;\;\;\;,t>t_{\lambda GIstart}\\ \lambda_{GI0}\;\;\;\;\;\;\;\;\;\;\;\;\;\;\;\;\;\;\;\;\;\;\;\;\;\;\;\;\;\;\;\;\;\;\;\;\;\;\;\;\;\;\;\;\;\;\;,\;\;\;t\leq t_{\lambda GIstart} \end{array} \end{array}$$ (15)

so that *t*_λ*GI*50_ is, for any value of *ν*_λ*GI*_, the time at which ${\tilde{\lambda }}_{GI}$ has decreased by 50% of $\lambda_{GI0}(1-f_{\lambda GI}^{min})$. This “natural” value of hepatic insulin sensitivity ${\tilde{\lambda }}_{GI}$ is also possibly modified by therapy to yield the actual current value λ_*GI*_, depending on therapy effect *f*_*exp*_(LyLamgiCurr, *α*_*LyLamgi*_, *β*_*LyLamgi*_, *t* − *t*_*thx*_).

#### Stomach emptying rate

*k*_*JS*_ is the rate expressing apparent first-order stomach emptying. In order to allow for the possibility that its value changes over time (e.g. it may be affected by drugs slowing gastric emptying), it is represented as a variable rather than a parameter. Its healthy constant average value is indicated by

$$\begin{array}{c} {\tilde{k}}_{JS}\;\;\;=\;\;\;k_{JS0}\; \end{array}$$ (16)

and may be modified by therapy to yield the actual current value *k*_*JS*_, depending on therapy effect *f*_*exp*_(LyKJS, *α*_*LyKjs*_, *β*_*LyKjs*_, *t* − *t*_*thx*_).

We therefore endowed the model with the ability to represent in general, for each treatment and for each affected control variable, arbitrary rates of onset of effect and arbitrary rates of loss of the same. In the current version of the model, the five control variables for which this scheme has been implemented are *η* (*β*-cell replication reserve restoration), *k*_*XGI*_ (peripheral insulin sensitivity), λ_*GI*_ (hepatic insulin sensitivity), *k*_*IB*_ (insulin secretory function)and *k*_*JS*_ (glucose absorption rate from the GI tract).

### Daily model

In order to describe the fast variations of glycemia over minutes, across a time span typically not exceeding the length of one day, a fast, “Daily” model is utilized. The present version of the fast model borrows heavily from previous rapid glucose-insulin control models, which proved to be effective and parsimonious representations of fast dynamics during acute perturbation experiments (both Intra-Venous and Oral Glucose Tolerance Tests, IVGTT and OGTT) [48–51]. As mentioned above, we denote “fast” time with the greek letter *τ* in order to underscore the difference between “fast” variables and slowly varying phenomena, which change over the months of “slow” time *t*, as in the previous equations.

#### Gastrointestinal glucose transit and absorption

The variation of *S*(*τ*), glucose content in the stomach, is described as a simple first-order linear elimination after impulsive loading corresponding to the meals:

$$\begin{array}{c} \frac{dS}{d\tau }=-k_{JS}\;S\;\;+\;\;\sum_{m=1}^{3}\delta (\tau \;-\;\tau_{m})\;M_{m}^{\text{gluc}}\;,S\left(\tau_{0}\right)=0 \end{array}$$ (17)

Similarly, the rate of change of glucose content in the intestine (indicated as “Jejunum”, J), based on delivery from the stomach and exit into the circulation, is expressed as a simple linear ODE:

$$\begin{array}{c} \frac{dJ}{d\tau }=k_{JS}\;S-k_{GJ}\;J,\;J\left(\tau_{0}\right)=0 \end{array}$$ (18)

The rate of glucose appearance in the systemic circulation, following intestinal absorption, is a fraction of the disappearance rate of glucose from the “jejunum” (since some of the absorbed glucose is either utilized or stored by the gut and/or liver):

$$\begin{array}{c} ra=\;f_{GJ}\;k_{GJ}\;J\;\; \end{array}$$ (19)

#### Fast plasma glucose concentration

It is first of all to be clarified that “Fast” is here meant as the opposite of “Slow” and not as characterizing an early morning or otherwise “fasted” state. The point here is that while some characteristic daily glucose concentrations, such as for instance early-morning fasting glycemia *G*_*f*_(*t*), vary over the months of a lifetime, glucose concentration *G*(*τ*) can also be thought of as varying, minute-by-minute, over the period of an experiment or over a given day in the life of the individual. The mass balance considerations determining the evolution of plasma glucose concentration over a given day are described by the following Equation, contemplating insulin-dependent glucose elimination (saturating), possible insulin-independent linear glucose elimination, and contributions to the variations of plasma glycemia deriving from liver gluconeogenesis and glycogen lysis, from the action of glucagon (L), from brain glucose oxidation, from the appearance of foodstuff-derived glucose, and from the loss of glucose in the urine:

$$\begin{array}{c} \begin{array}{cc} \frac{dG}{d\tau }= & -k_{XGI}^{max}\;\;\frac{I}{I\;\;+\;\;I_{KXGI50}}\;\;G\;\;-\;\;k_{XG}\;G\;\\ & +\frac{1}{V_{G}W}(\;k_{GI}^{max}\;e^{-\lambda_{GI}IG}\;+\;\;k_{GL}^{max}\frac{L}{L_{G50}+L}-W\;\;k_{Xg\;brox}^{max}\frac{G}{G_{brox\;50}+G}\;\;+\;\;\text{ra}\;\;-\;\;\text{ur})\;;\;\;\\ G(\tau_{0}) & =G_{f}(t) \end{array} \end{array}$$ (20)

At any slow time *t*, we assume we start our daily evolution from a fasting glycemia value *G*_*f*_(*t*). Peripheral insulin sensitivity depends on the prevailing levels of insulin (in the sense that the marginal utility of an increase in insulin decreases as levels of the hormone increase). When considering tissue insulin sensitivity to be nonlinearly increasing with increasing insulin concentration, we wish in any case to retain the common notion of a (linear) insulin sensitivity index at zero insulinemia, $k_{XGI}^{0}$, with the same measurement units and with comparable numerical values with respect to previously published insulin sensitivity indices (see Panunzi *et al*. [50] for a comparative discussion of two such indices). In the present formulation, $k_{XGI}^{0}$ expresses the slope of the non-linearly increasing peripheral tissue insulin sensitivity at *I* = 0. Its value should be around 1.*e* − 4[/*min*/*pM*] for normal individuals [48, 50] and somewhat higher for fit, athletic individuals. At a value $k_{XGI}^{0}\;\;\;=\;\;\;1.e-4\;/min/pM$ and with a reasonable insulinemia at half-effect $I_{KXGI50}\;\;\;=\;\;\;\frac{k_{XGI0}^{max}}{k_{XGI}^{0}}\;\;\;=\;\;\;500\;pM$ [52] we find a current value $k_{XGI0}^{max}\;\;\;=\;\;I_{KXGI50}\;k_{XGI}^{0}\;\;=\;\;\;500\;\cdot \;\;1.e-4\;\;\;=\;\;\;0.05\;/min$, which seems very reasonable (i.e. maximum tissue uptake of glucose at hyperinsulinization, or in other words maximal clearance of glucose from plasma at extremely high levels of insulin, is approximately 5%/*min*).

We could directly extend the model by considering different meal compositions at breakfast, lunch and dinner, with ensuing differences in gastric emptying rate and glucose absorption rate. This could be attained by considering *N*_*foods*_ types of foodstuff indexed by *j* at each meal *m*: different foodstuffs would load separate *S*_*j*_ and *J*_*j*_ compartments, with transit governed by $M_{j}^{gluc}$, *f*_*GJj*_, *k*_*GJj*_, *τ*_*j*_, and *k*_*JSj*_ parameters. For simplicity, the uniform meal composition formulation is retained. The **ur** renal glucose elimination rate is provided by the concurrently running nephron model (see below).

#### Fast serum glucagon concentration

The fast model contemplates glucagon as an index of the overall counterregulatory response.

$$\begin{array}{c} \frac{dL}{d\tau }=-k_{XL}\;L+\;\;k_{LG}^{max}\;\;(f_{{}_{LG}}^{min}\;+\;(1-f_{{}_{LG}}^{min})e^{-\lambda_{LG}\;G}),\;L\left(\tau_{0}\right)=L_{0}\; \end{array}$$ (21)

Glucagon plasma concentration (L) is supposed to undergo first-order linear elimination [53, 54]. This has in fact been demonstrated to be true in man [53] as well as in dogs, with a suggestion that the process may be saturable at pharmacologic concentrations [55]. Glucagon plasma concentration has also been shown to increase above some minimum (determined by some measure of continuous production), in an exponentially increasing fashion as glycemia decreases: a large set of experimental data were fitted to show this relationship [56]. We note that there is literature support for the idea that insulin cannot override hypoglycemia in suppressing glucagon secretion [57] (Cavallo-Perin *et al*. [58] showed only 20 − 40% suppression of fasting glucagon levels with euglycemic clamps when plasma insulin was increased to 350 pM, and similarly Elahi *et al.* when using 700 pM [59]). A determinant role for insulin would seem to be indicated by the observation of hyperinsulinemic coma with low glucose and glucagon concentrations: the present form of the model has however been considered synthetic and sufficient to describe relative insulin deficiency situations, such as occur with the development of T2DM. Clearly, insulinomas and similar conditions would be poorly represented by the current form of Eq 21. Notice also that we assume the dynamics of glucagon to remain unchanged over slow time. Even so, there are situations [60] where glucagon release is substantially reduced in T1DM, though the mechanisms for this are not entirely clear [61].

#### Fast serum insulin concentration

Insulin kinetics in the short period may be approximated, following mass-balance considerations as

$$\begin{array}{c} \frac{dI}{d\tau }=-k_{XI}\;I+\frac{\psi_{I}^{gluc}\;(B,[G(1+\;f_{IJ}\;\frac{J}{J_{G50}+J})\;]\;)}{V_{I}\;W}\;\;,\;\;\;\;\;I\left(\tau_{0}\right)=I_{f}(t)\; \end{array}$$ (22)

with $\psi_{I}^{gluc}$ given by Eq 8.

Insulin is assumed to be eliminated from plasma in a first-order, linear fashion. The entry of insulin into plasma derives from glucose-driven, saturable pancreatic secretion. For the purpose of the present model we do not distinguish peripheral from portal insulin concentration, therefore hepatic insulin action will be made to depend on peripheral insulin concentration itself (whose effect will be apparently larger with larger hepatic insulin extraction). The equations above express insulin secretion as depending linearly on the currently available *β*-cell mass and nonlinearly, in a saturable way, on glycemia. Notice that, in order to represent incretin contribution to the stimulation of insulin secretion, the effectiveness of glycemia is supplemented (saturably) by current intestinal glucose contents, via a proportionality constant *f*_*IJ*_.

### Nephron model

The concept of Renal Glucose Threshold is well rooted in common medical and diabetological practice. According to this concept, renal elimination of glucose occurs when, glycemia having exceeded some threshold *G*_*thresh*_, glucose delivery to the nephrons exceeds their reabsorptive ability. While physiologically very plausible, the concept has unfortunately been translated, typically, into a mathematical formulation stating that if at some time *τ* glycemia *G*(*τ*)>*G*_*thresh*_, then at that time there will be glucose loss in the urine at a rate proportional to the difference *G*(*τ*) − *G*_*thresh*_. The problems arising from this simplistic interpretation of the glucose threshold principle have been treated in detail elsewhere [62]: in the same work, an alternative, partial differential formulation of the principle, has been proposed, which does not suffer from the problems described. In the current context we therefore use the same partial differential equations approach used in that publication in order to build a simple nephron sub-model, able to realistically represent glucose elimination in the urine produced by glycemia oscillations over time. For the reasons explained in detail in the work referred to above [62], we believe that the simpler renal-threshold sub-model would predict glucose renal elimination inappropriately over the very glycemic range around the supposed threshold, range that is repeatedly entered by glucose concentrations in the course of the day, particularly in insulin-resistant subjects. Having a more advanced nephron sub-model available, we took advantage of it, incorporating it in the overall disease progression model. It is to be noticed that in the nephron model the considered time *τ* in minutes is the same “fast” time as in the Daily model above: in fact, the Nephron and Daily sub-models progress in parallel through a typical short time period (a few hours), with the Daily model determining at each discretization step the current glycemia, used by the Nephron model to determine at each step the corresponding urinary elimination, used then again by the Daily sub-model to determine glycemia variations and the resulting glycemia, and so on.

#### Density *q* of glucose at time *τ* at nephron level *z*

The nephron model considers amounts of glucose in small (infinitesimal) segments of an idealized single tubule, representing the collection of glucose-reabsorbing proximal and distal nephron tubular sections. Henceforth, all quantities are to be understood as referred to Kg body weight. The density *q* of glucose quantity *Q* with respect to normalized tubule length *z* (*z* ∈ [0, 1]) varies over time and along the tubule, *q* = *q*(*τ*, *z*):

$$\begin{array}{c} \begin{array}{c} \frac{dq}{d\tau }\;\;\;=\;\;\;D_{U}\;\;\frac{\partial^{2}\;C\;}{\partial \;z^{2}}\;\;-\;\;\phi_{U}(z)\frac{\partial \;C\;}{\partial \;z}\;\;-\;\;\frac{k_{GU}^{max}}{1}\;\lambda_{QZ}\;e^{-\lambda_{QZ}z}\frac{C}{C_{GU50}\;\;+\;\;C}\;\;\;\;,\\ q(0,z)\;\;\;=\;\;\;G_{0}\;v(z)\;\;e^{-\lambda_{QZ}z}\;\;\;,\\ q(\tau ,0)\;\;\;=\;\;\;G(\tau )\;v(0)\;\;\;. \end{array} \end{array}$$ (23)

The variation of glucose density over time at some point *z* in the tubule is given in general by a transport equation with diffusion *D*_*U*_ along the tubule, with advection driven by the flow-rate *ϕ*_*U*_ (variable along the tubule), and by saturable extraction from the tubule lumen operated by lining cells. All three effects are expressed in terms of concentration *C* = *Q*/*V* = *q*/*v*. The notation $\frac{k_{GU}^{max}}{1}$ indicates that the maximum total transport by the tubule is to be divided by the total z-length of the tubule itself, which in the present normalized case equals exactly 1. We further assume that glucose reabsorptive capacity is essentially zero at the end of the tubule, so that $e^{-\lambda_{QZ}\cdot 1}\approx 0$ and $\int_{0}^{1}\lambda_{QZ}\;e^{-\lambda_{QZ}}\;z\;dz\approx 1$, so that $k_{GU}^{max}$ does indeed represent the maximum glucose absorptive capacity of the entire tubule system.

In this formalization, the volume density *v* (akin to the total tubular cross-sectional area at some level in the tubular system) is defined as the density of water amount with respect to normalized tubule length, reflecting progressive water reabsorption along the nephron, and is proportional to 180 L/day ultrafiltration at *z* = 0 and to 2 L/day urine output at *z* = 1 (in a 70-kg person). An approximation to the volume density profile *v*(*z*) at given depths down the renal tubules is obtained by hypothesizing an exponential volume decay over the length of the tubule, with entry proportional to the flow of ultrafiltrate and exit proportional to the flow of urine:

$$\begin{array}{c} v(z)\;\;\;=\;\;\;\frac{(\text{ClInulin}\;\;-\;\;\text{Furine})\;\;e^{-\lambda_{VZ}z}\;\;+\;\;\text{Furine}}{r} \end{array}$$ (24)

where *r* is the apparent normalized rate of movement of ultrafiltrate along the tubule, set to $r=\frac{1}{T}$, with *T* the hypothesized time of permanence of ultrafiltrate in the tubules. With this notation, the flow-rate *ϕ*_*U*_(*z*) is simply *ϕ*_*U*_(*z*) = *rv*(*z*). Notice that for λ_*VZ*_ sufficiently large, *ϕ*_*U*_(1) is arbitrarily close to **Furine**.

The initialization profile *q*(0, *z*) is only a first rough (exponentially decaying) approximation to the quantity of glucose in tubular water: before starting with the Daily / Nephron numerical integration, the Nephron model is run at *G* = *G*_0_ for as long as necessary to reach convergence in the *q* profile. The boundary condition *q*(*τ*, 0), on the other hand, is given by the glucose concentration in plasma at time *τ* times the volume density at *z* = 0.

Given quantity and volume, the concentration *C* of glucose in the pre-urine is algebraically determined:

$$\begin{array}{c} C(\tau ,z)=\;\;\;\frac{q(\tau ,z)}{v(z)} \end{array}$$ (25)

Finally, the rate of glucose elimination in the individual at time *t* is given by the most distal/caudal (pre-)urine glucose concentration (i.e. glucose concentration at *z* = 1) times the total urinary flow rate:

$$\begin{array}{c} \text{ur}(\tau )\;\;\;=\;\;\;\;W\;\;\cdot \;\;C(\tau ,1)\;\;\cdot \;\;\text{Furine} \end{array}$$ (26)

### Index variables

One fundamental assumption of the present model is that the variation over months and years of the “slow” homeostasis variables (such as *β*-cell population size or pancreatic *β*-cell replication reserve) may depend not only on monthly averages of fast variables (such as glycemia or insulinemia), but also on other slow functions of these same fast variables, possibly derived from the explicit computation of the time course of the fast variables over a representative day. Examples of these slow indices derived from fast dynamics and possibly affecting disease progression are average glycemia, glycemic variability, or post-meal glycemic peaks. Commonly used clinical indices such as the HOMA-IR or the EHC M-values are also computed as slow index variables. The weighted daily glucose toxicity determining *η* suppression, as fraction of normal (normal = 100% at fixed 5.5 mM glycemia) derived from the TUNEL [37] study, also belongs to this class. The definition of some such index variables is straightforward, assuming the daily time course of the necessary fast variables to be available: mean daily glycemia and insulinemia; their standard deviations, minima and maxima; their value at relevant times (e.g. baseline, 30 min, 60 min, 2 hours after administration of the glucose bolus during an Oral Glucose Tolerance Test). Given the availability of the necessary ingredients, the computation of some clinically relevant slow indices (HOMA-IR, HOMA-B, insulinogenic Index) is also straightforward. Finally, a two-step Euglycemic Hyperinsulinemic Clamp experiment (120 min at 100 pM insulinemia, followed by 120 min at 420 pM insulinemia, replicating conditions that have been used previously to assess respectively hepatic and peripheral insulin sensitivity) can be conducted on the virtual subject at any time, and the corresponding low- and high-insulinization Glucose Infusion Rate (GIR) values, expressed in mg/kgBW/min are also slow indices of interest and are indicated as ClampM1 and ClampM2.

### Parameter assessment

The model proposed here combines disease progression over slow time with daily absorption, metabolism and renal elimination in fast time. Parameter values for the slow time component have been derived from the in-depth assessment undertaken with the publication of the previous model [10] wherever applicable. This includes fasting glycemias and insulinemias; deduced characteristics of *β*-cell replication (but see also [18] and above comments on the switch from linear to saturable model of *β*-cell population dynamics); pancreatic *β*-cell replication reserve capacity; glucose toxicity; insulin secretion (per millon *β*-cells) and elimination rates; glucose effectiveness; production and decay rates of glycosylated haemoglobin. However, the model introduces separate insulin sensitivity components for hepatic (and possibly renal) and peripheral (mainly referred to muscle and adipose tissue) insulin sensitivity, and assumes that both components may vary over slow time (years or decades) depending on genetics, lifestyle, intervening diseases etc.

Furthermore, insulin independent glucose clearance (mostly due to brain glucose consumption) has been explicitly introduced in the fast glycemia equation.

Parameter value assessment, unless specifically discussed below, follows the same approach as delineated for the previous model [10]. The majority of parameters were obtained from in vivo studies on adult subjects, pediatric or developmental data were not considered. Typical values and ranges at time *t*_0_ were generally taken from data obtained in young, healthy adults (age 18-30 years): while *t*_0_ is defined as the time of birth for the present work, we make no attempt to actually replicate the physiological variations of the glucose-insulin system along infancy and childhood. Rather, we let simulations through the first 18 years of age run on early adulthood parameters, thereby reaching a normal or healthy steady state at age 18, and start perturbing the model (e.g by imposing a progressive decrement in insulin sensitivity) after age 18 years.

Parameters describing the average evolution of insulin resistance in the whole patient population were calibrated in order to achieve starting conditions at age 50 consistent with the overall average starting conditions in the DPP study, where average age at enrollment was approximately 50 years. More specifically, we simulated an average subject presenting as pre-diabetic at age 50 by introducing a progressive decline (after age 18) in peripheral and hepatic insulin sensitivity, as well as a modest, progressive decline in insulin secretory function (see the functional description of *k*_*XGI*_, λ_*GI*_ and *k*_*IB*_ in Eqs 15, 14 and 12, Table 2).

The next step was to calibrate those parameters describing the rates of onset and decay and the size of the effects produced by the three experimental maneuvers considered (Placebo, Metformin, LifeStyle), based on our understanding of the likely physiological effect of the three treatments, in order to reproduce observed time courses for fasting and post-prandial glycemias (Table 3). We assumed, in particular, that the DPP treatments would likely impact hepatic and/or peripheral insulin sensitivity and would not have a direct effect on insulin secretion; moving from this assumption, by changing the rapidity of onset, rapidity of decay and size of the hepatic and peripheral insulin sensitivity improvements for each treatment, we explored the ability of the model to reproduce simultaneously the size and shape of the observed time courses of fasting and post-prandial glycemias and insulinemias.

The selection of parameter values was subjective (visual assessment). While a large number of simulations were performed, no systematic exploration of the whole (high-dimensional) parameter space was conducted, but rather the parameter value combinations reflecting our understanding of the likely effects of the various interventions were marginally adjusted to better approximate the data points. For example, when considering the adaptation of the model predictions to the observed DPP averaged data points, we had to improve peripheral insulin sensitivity, over predicted no-intervention levels, by a maximum of about 10% for Placebo and Metformin and 22% for the Intensive LifeStyle group; further, while rates of decay of effect were apparently similar for the three groups, onset of effect was much faster for Intensive LifeStyle than for Placebo or Metformin.

In the present work the emphasis was on showing the reasonableness and robustness of the model rather than on estimating effect size. Since the model is relatively large with respect to the independent sources of information from the DPP study (which, together with likely correlations among parameters, would have made the model *a-posteriori* unidentifiable), we eschewed the use of formal optimization of some classical loss function for statistical parameter estimation. While the model structure is apparently correct in that it can replicate observations, we cannot therefore assess the variability of the parameter estimates, cannot construct confidence intervals around them and cannot offer measures of Goodness-of-Fit.

### DPP dataset

The objectives and results of the Diabetes Prevention Program (DPP) study have been extensively described elsewhere [20, 21] Briefly, in this study the primary aim was that of evaluating the incidence of T2DM in an at-risk population randomized to placebo (n = 1082), intensive lifestyle modification (n = 1079), metformin (n = 1073), or troglitazone (n = 585). From 1996 to 1999, the study enrolled adult subjects with elevated Fasting Plasma Glucose (FPG from 5.6 to 7.7 mmol/L before June 1997; FPG from 5.3 to 6.9 mmol/L after June 1997) and 2-hr-Glucose (G2h from 7.8 to 11.0 mmol/L) during a 75g Oral Glucose Tolerance Test (OGTT), as well as elevated Body Mass Index (BMI ≥ 22 kg/m in Asians, BMI ≥ 24 kg/m otherwise). We obtained the original dataset from the DPP study through application from NIH-NIDDK (February 2008 Full Scale data release; data request No.608, see *Acknowledgements*). The data were fully anonymized as supplied by NID-NIDDK, not only was identifying information eliminated from the data set, but also subjects’ ages were not reported as recorded, but only in five-year classes. We excluded from this dataset a small number (59) of patients who presented with FPG in the diabetic range at entry into the study (“*Entry Diabetics*”) and proceeded then to compute averages of FPG (*G*_*f*_) at entry and at each six-monthly observation thereafter, as well as averages of 30-minutes and 2-hour glycemia (*G*_30*m*_, *G*_2*h*_) and baseline (FSI) and 30-minutes insulinemia (*I*_*f*_, *I*_30*m*_) after Oral Glucose Tolerance Test (OGTT), at entry into the study and at each yearly interval thereafter.

We did not receive any special access or privileges to the data: interested researchers will be able to access the data in the same manner as we did. Interested researchers can replicate our study findings exactly and in their entirety by implementing the equations constituting the model described in the Methods section, populating the implementation with the parameter values reported in the Tables; and finally plotting the resulting model predictions together with the averages of the DPP study data at each time point.

### Implementation

The computational engine of the model has been implemented in C++ (Microsoft^®^ Visual Studio Community Edition 2017), with a MATLAB^®^ graphical front-end (MATLAB version R2009b, The MathWorks Inc.). The model engine is also accessible both for guest researchers use (through a browser HTML interface) and as a web-service for guest machine-to-machine use (via a WSDL) at the address biomatlab.iasi.cnr.it/models/login.php.

## Results

**Fig 1:**
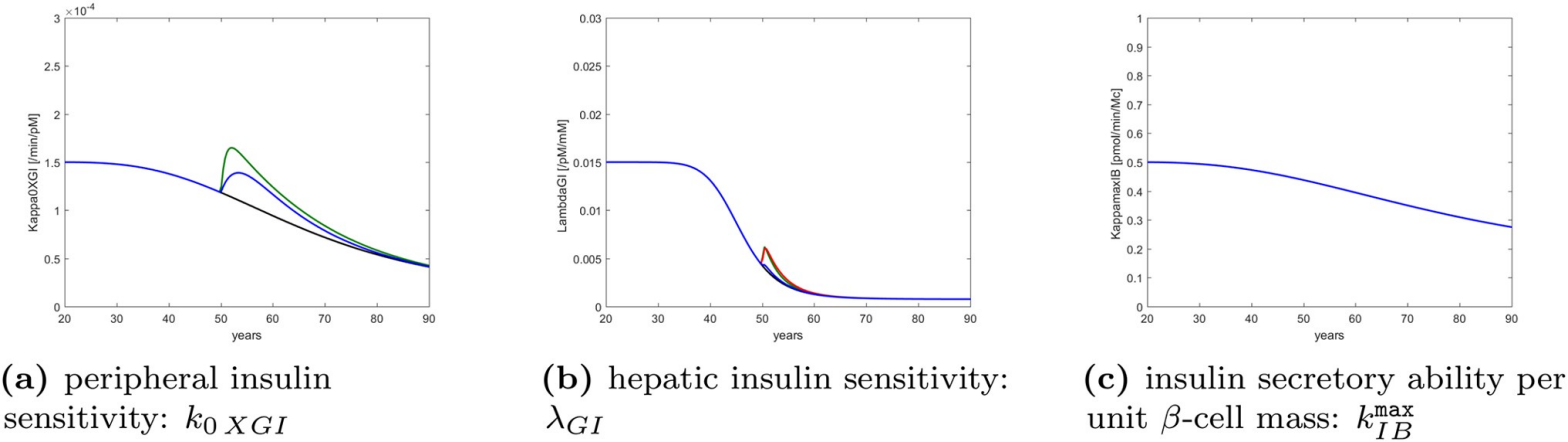
Hypothesized disease progression. Time course of parameters indicative of peripheral insulin sensitivity, hepatic insulin sensitivity and insulin secretory ability over the lifetime of a representative DPP study subject, as determined by genetic factors, alimentary habits and other life conditions. Black refers to the natural course of the disease, blue to intervention with Placebo, green to intervention with Intensive Life-Style modification, red to intervention with Metformin. In panel (a) the Metformin curve is identical with Placebo; the four forecasts are identical for insulin secretory ability in panel (c).

Fig 1 shows the hypothesized disease progression in terms of the evolution of peripheral insulin sensitivity (Panel **a**, substantially decreasing with age), hepatic insulin sensitivity (Panel **b**, substantially decreasing around ages 40 to 60), and insulin secretory ability per unit *β*-cell mass (Panel **c**, mildly decreasing with age). This is a combination of original modifications (attributable to advancing age, lifestyle factors and dietary habits), which can explain, through the model, the observed average changes in all three treatment arms.

**Fig 2:**
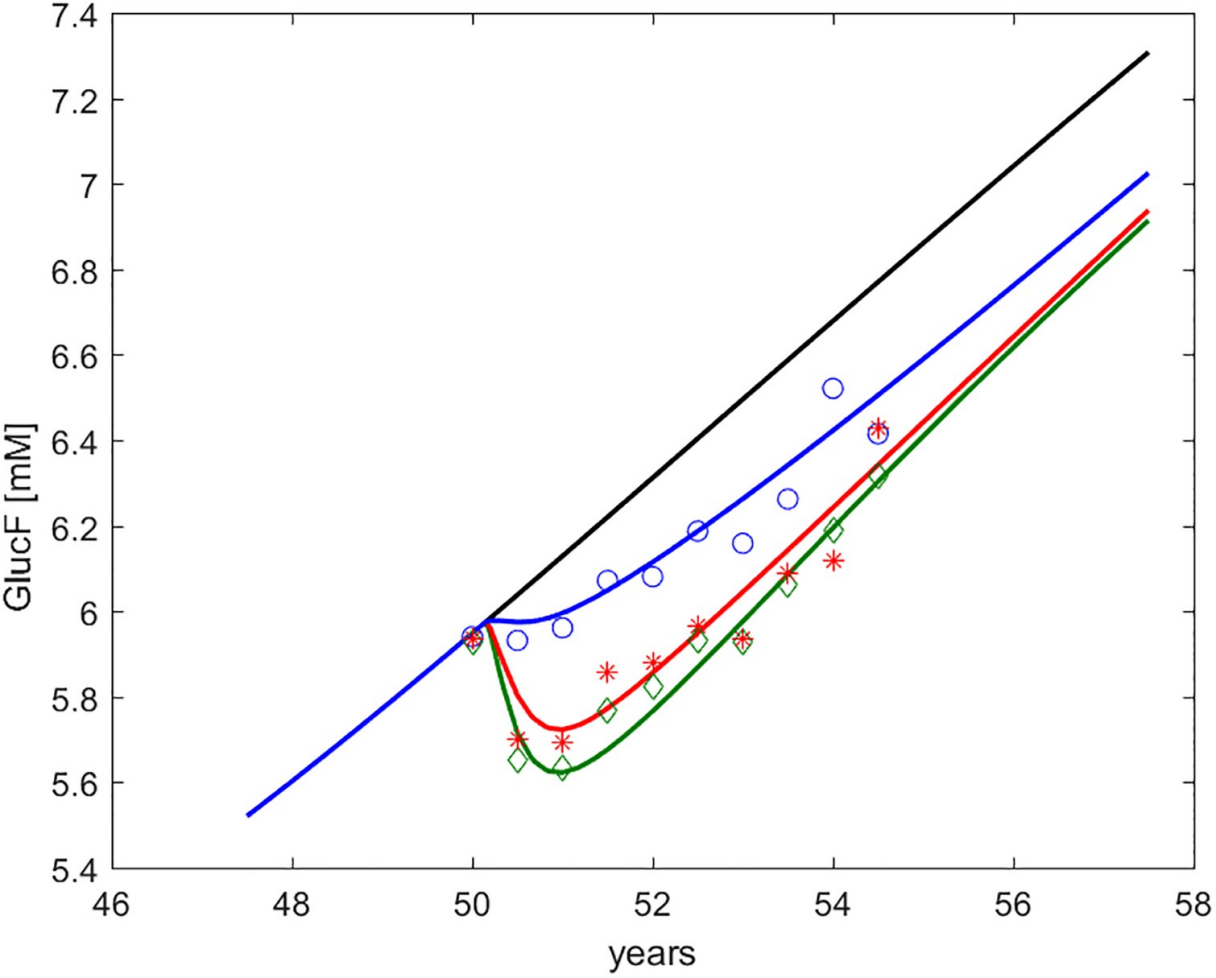
Fasting Plasma Glucose (*G*_*f*_ or FPG, indicated as GlucF). Time course of predicted and observed average Fasting Plasma Glucose, around the period of study. The four curves refer to no intervention (black), Placebo (blue), Metformin (red), Intensive LifeStyle (green). The data points refer to Diabetes Prevention Program (DPP) study means for Placebo (blue circles), Metformin (red asterisks) and Intensive LifeStyle modification (green diamonds).

Figs 2 through 6 show the time course, over an interval of slow time spanning the study period, of the endpoints measured in the DPP study, together with the computed time-courses of the corresponding model variables.

**Fig 3:**
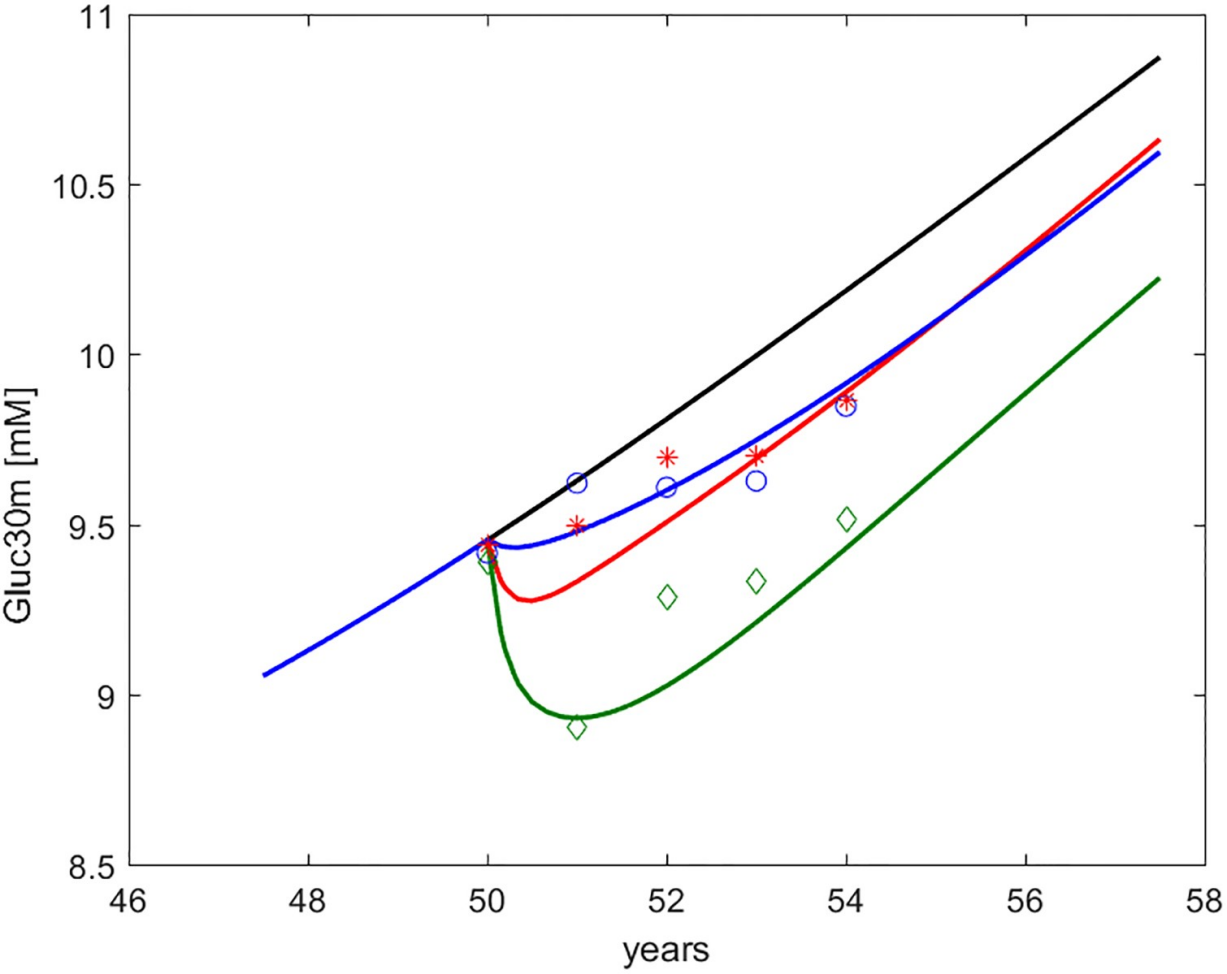
Glycemia at 30’ during OGTT (*G*_30*m*_, indicated as Gluc30m). Time course of predicted and observed average glycemia at thirty minutes during OGTT, around the period of study. The four curves refer to no intervention (black), Placebo (blue), Metformin (red), Intensive LifeStyle (green). The data points refer to Diabetes Prevention Program (DPP) study means for Placebo (blue circles), Metformin (red asterisks) and Intensive LifeStyle modification (green diamonds).

**Fig 4:**
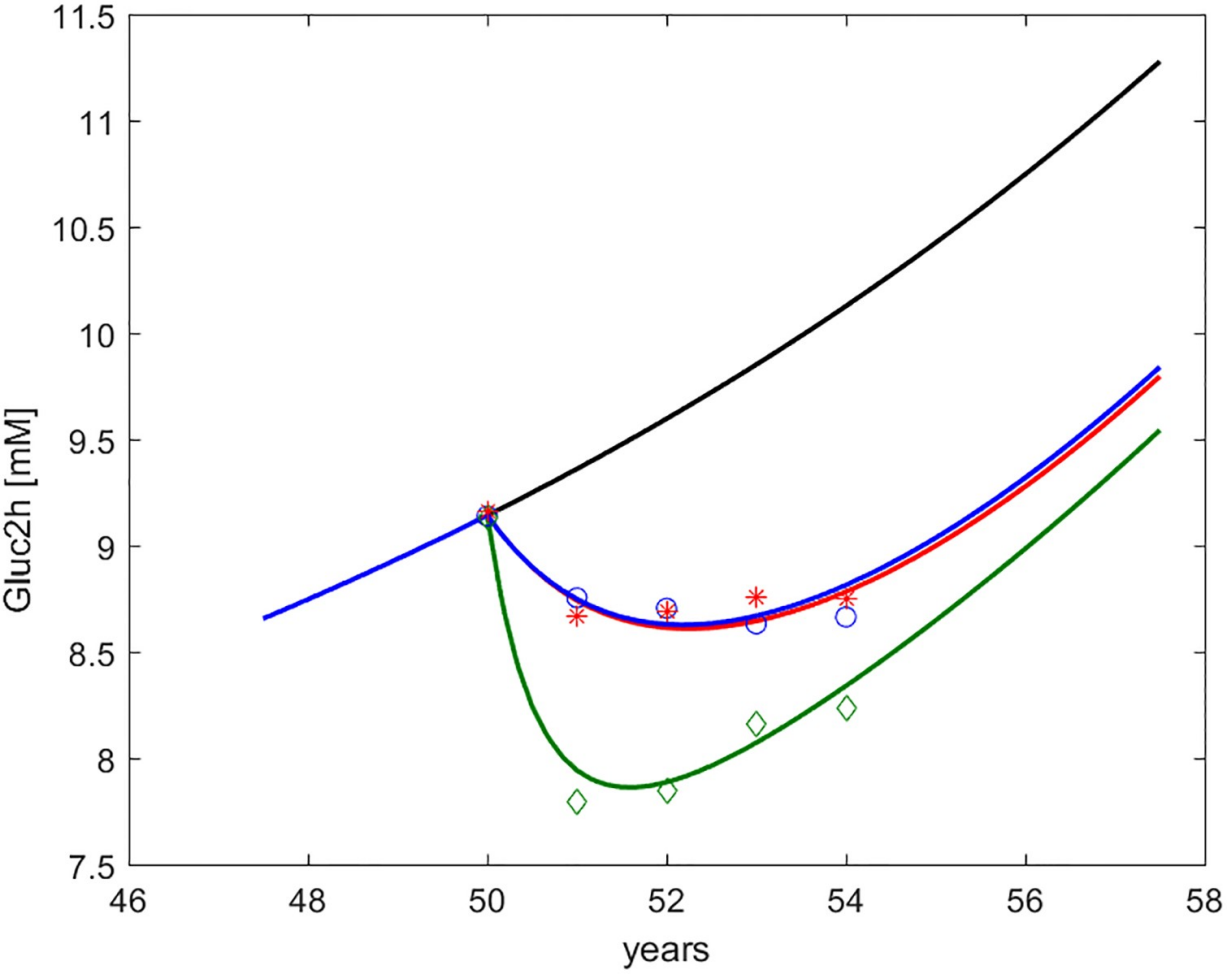
Glycemia at 2h during OGTT (*G*_2*h*_, indicated as Gluc2h). Time course of predicted and observed average glycemia at two hours during OGTT, around the period of study. The four curves refer to no intervention (black), Placebo (blue), Metformin (red), Intensive LifeStyle (green). The data points refer to Diabetes Prevention Program (DPP) study means for Placebo (blue circles), Metformin (red asterisks) and Intensive LifeStyle modification (green diamonds).

**Fig 5:**
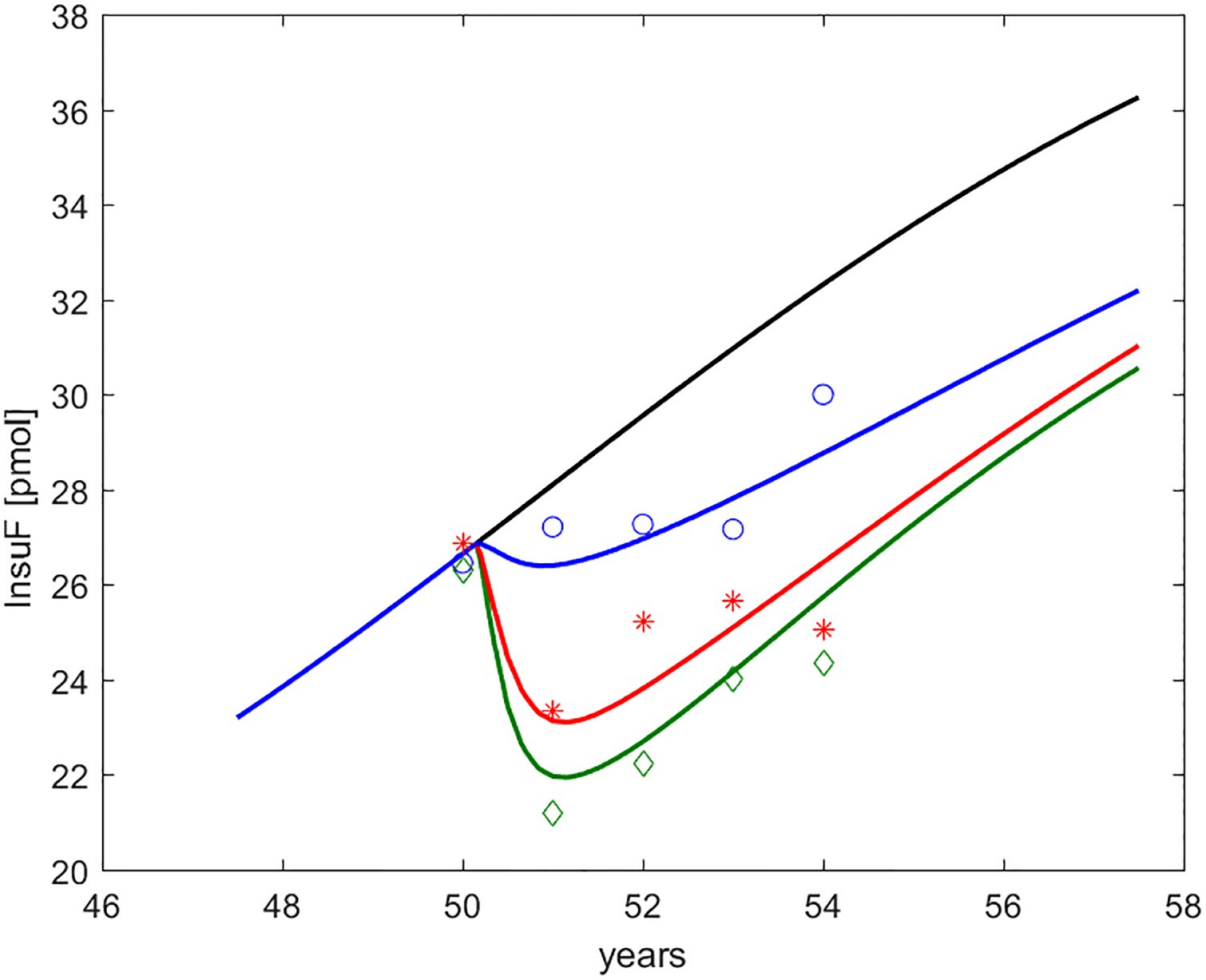
Fasting serum insulin (*I*_*f*_ or FSI, indicated as InsuF). Time course of predicted and observed average fasting serum insulin around the period of study. The four curves refer to no intervention (black), Placebo (blue), Metformin (red), Intensive LifeStyle (green). The data points refer to Diabetes Prevention Program (DPP) study means for Placebo (blue circles), Metformin (red asterisks) and Intensive LifeStyle modification (green diamonds).

**Fig 6:**
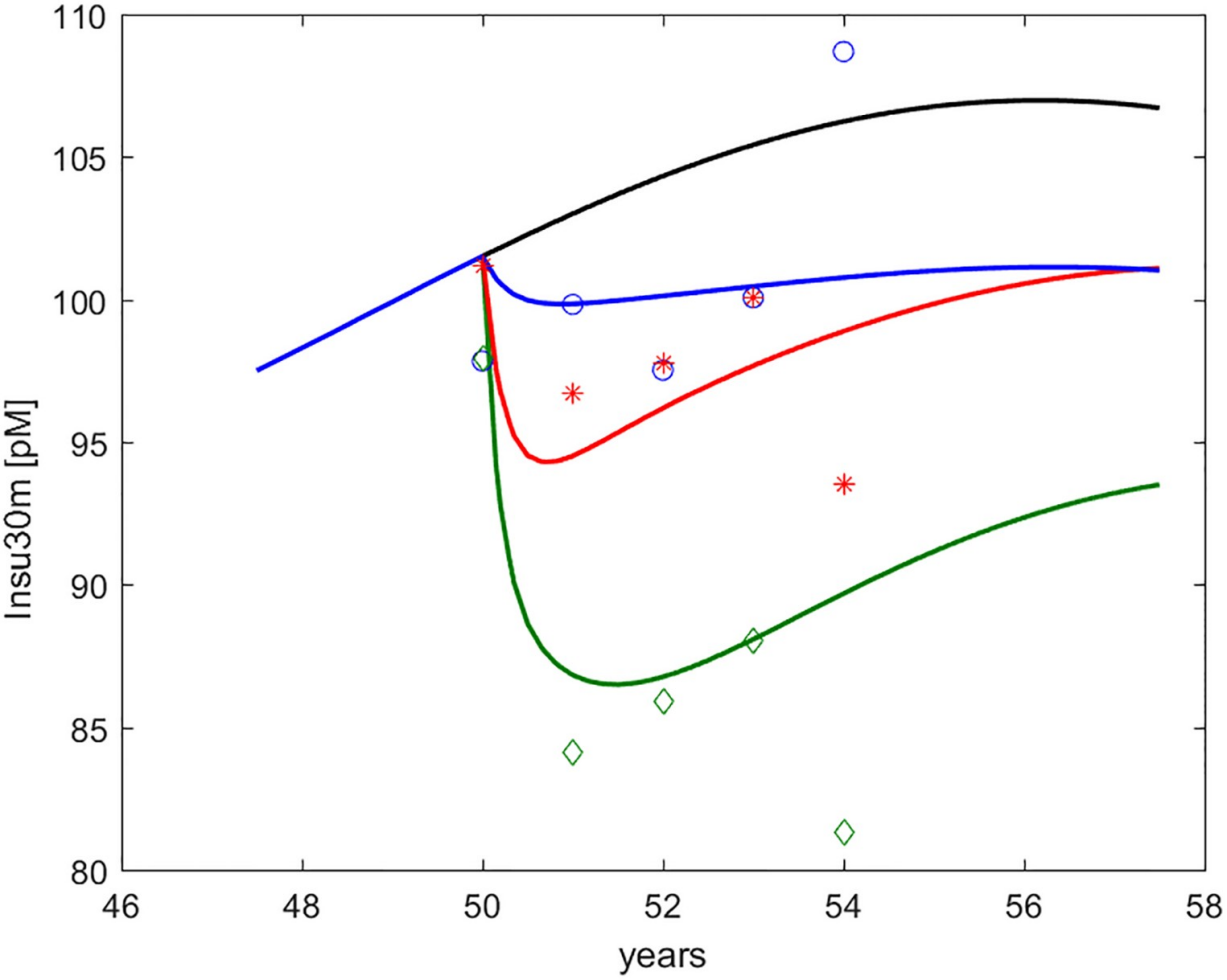
Insulinemia at 30’ during OGTT (*I*_30*m*_, indicated as Insu30m). Time course of predicted and observed average Serum Insulin at 30 minutes during OGTT, around the period of study. The four curves refer to no intervention (black), Placebo (blue), Metformin (red), Intensive LifeStyle (green). The data points refer to Diabetes Prevention Program (DPP) study means for Placebo (blue circles), Metformin (red asterisks) and Intensive LifeStyle modification (green diamonds).

**Fig 7:**
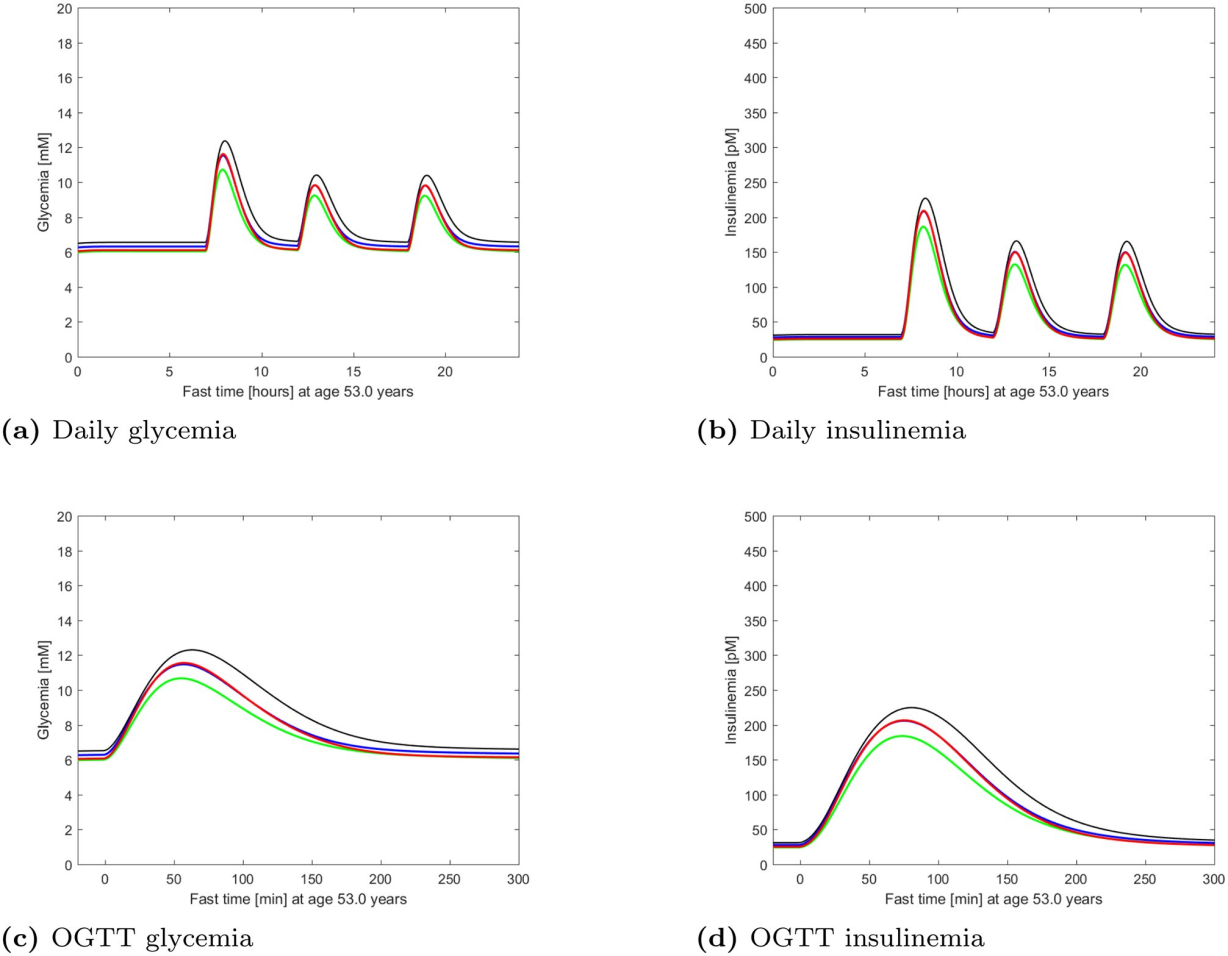
Fast glycemia and insulinemia. Time course of glycemia and insulinemia as predicted by the model for a representative virtual DPP subject, studied at age 53, during a simulated whole day (top panels) and a simulated OGTT (bottom panels). The curves refer to no intervention (black), Placebo (blue), Metformin (red), Intensive LifeStyle (green). Notice how the glycemic curve under Metformin tends to coincide with Intensive LifeStyle at fasting and with Placebo under glucose load.

Fig 7 shows daily and OGTT time courses of both glycemia and insulinemia as predicted by the model for a representative virtual DPP subject. From these, relevant clinical indices can be computed.

**Fig 8:**
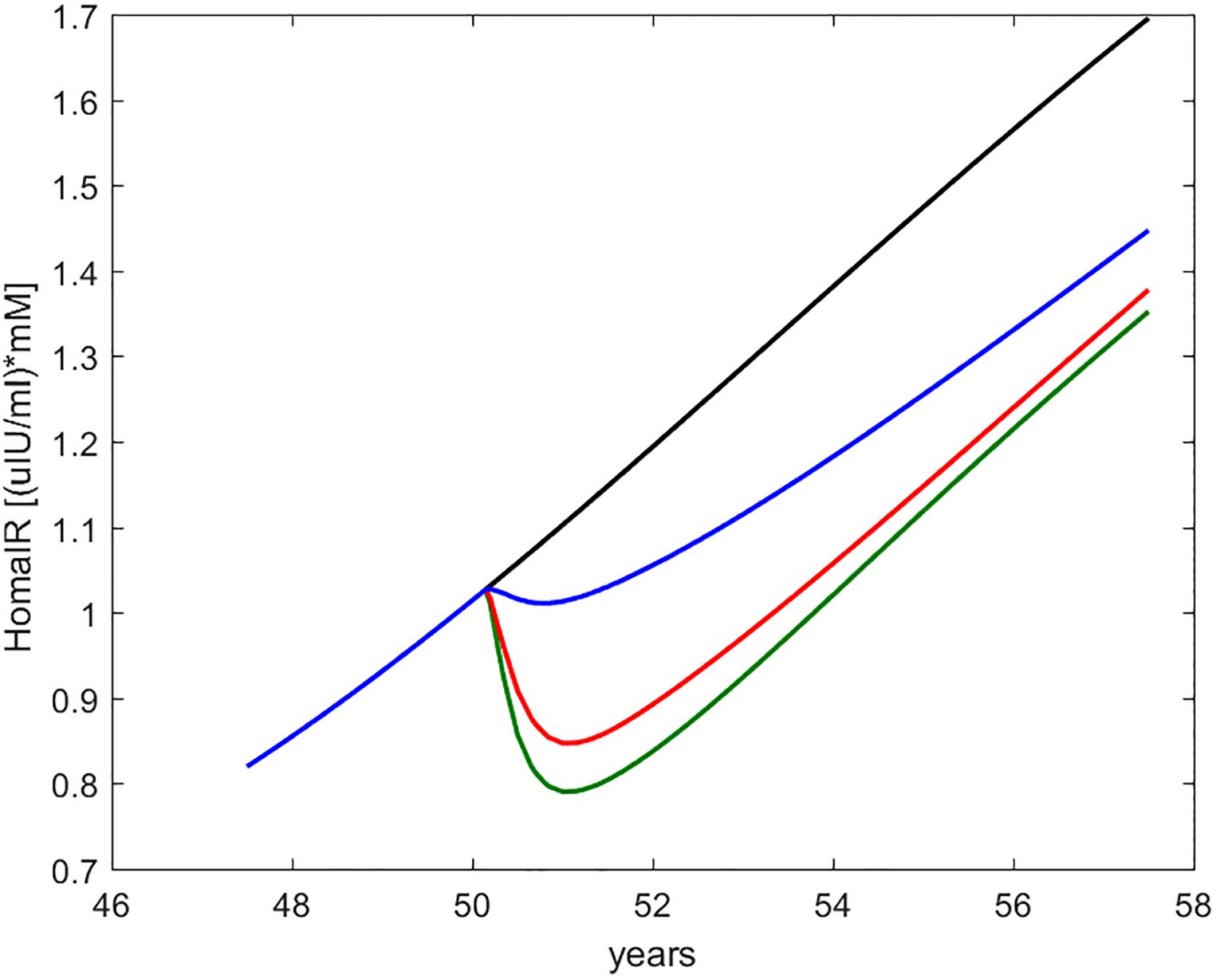
HOMA-IR. Time course of the HOMA-IR index as computed from current fasting glycemia and insulinemia values. The curves refer to no intervention (black), Placebo (blue), Metformin (red), Intensive LifeStyle (green).

Figs 8 through 12 show the time course, over the interval of slow time spanning the study period, of some commonly employed clinical indices of diabetic (de-)compensation: HOMA-IR, HOMA-B, insulinogenic index and 1^*st*^ and 2^*nd*^ phase clamp M indices.

**Fig 9:**
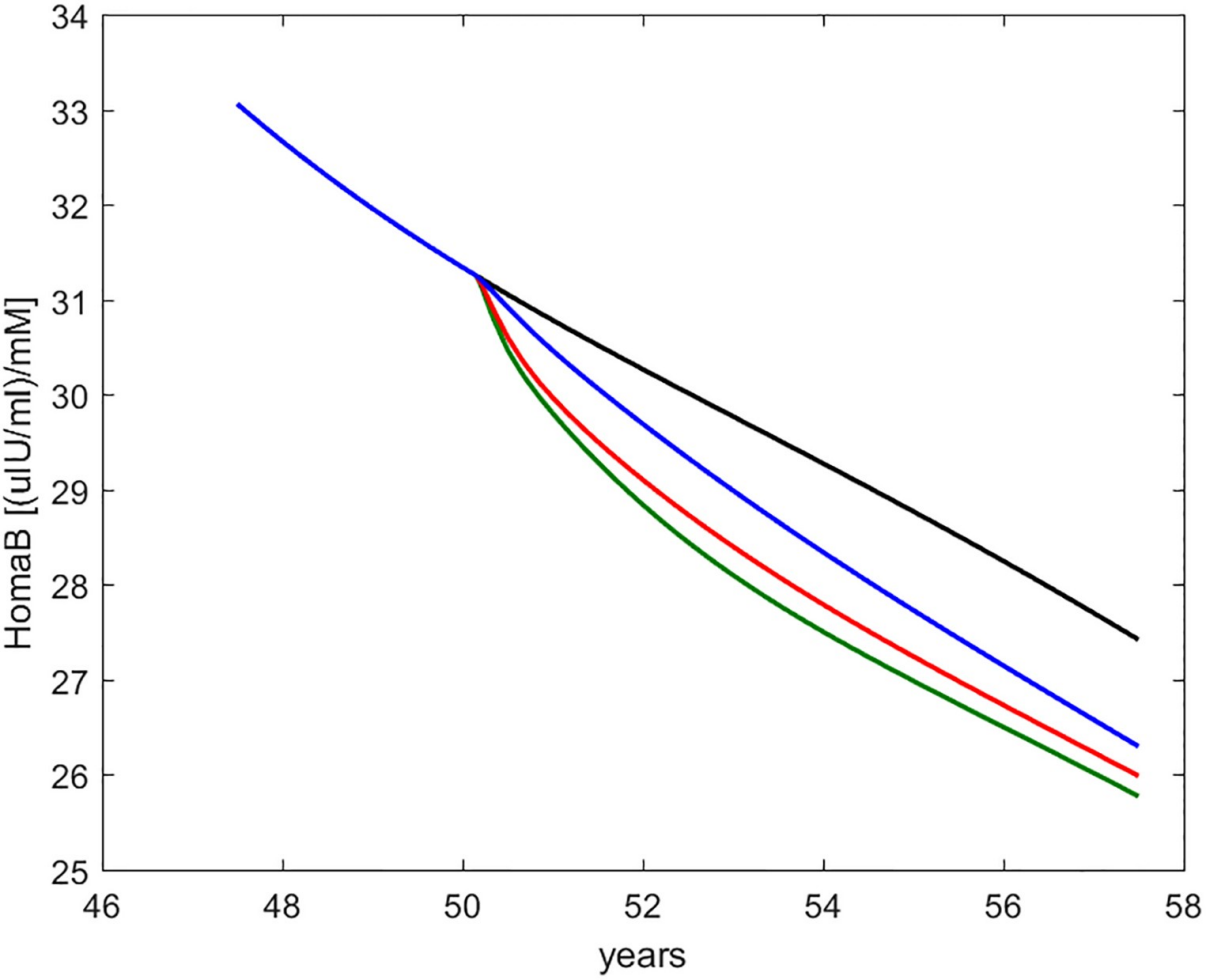
HOMA-B. Time course of the HOMA-IR index as computed from current fasting glycemia and insulinemia values. The curves refer to no intervention (black), Placebo (blue), Metformin (red), Intensive LifeStyle (green).

**Fig 10:**
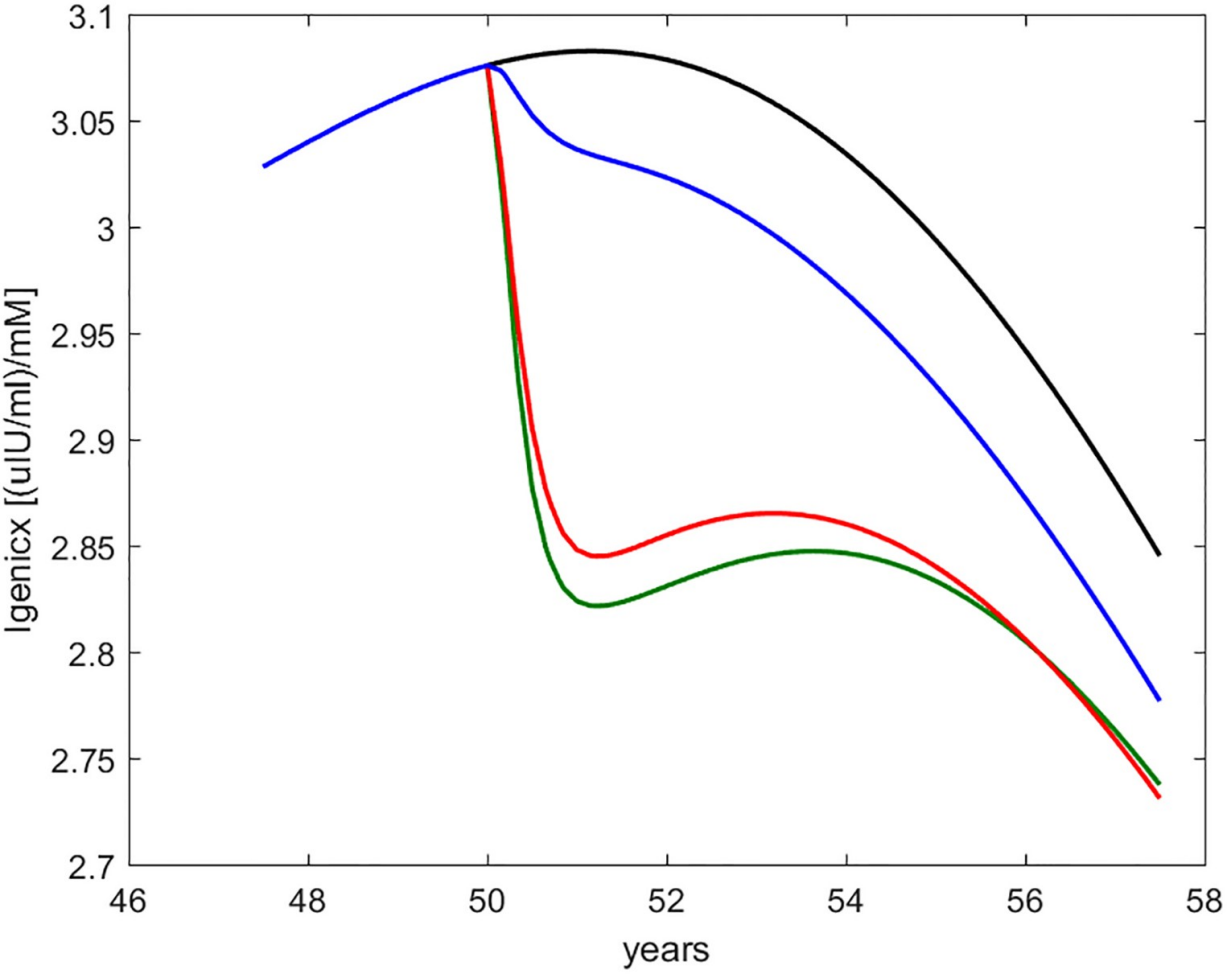
Insulinogenic index (indicated as Igenicx). Time course of the insulinogenic index as computed from current fasting and 30-min glycemia and insulinemia values. The curves refer to no intervention (black), Placebo (blue), Metformin (red), Intensive LifeStyle (green).

**Fig 11:**
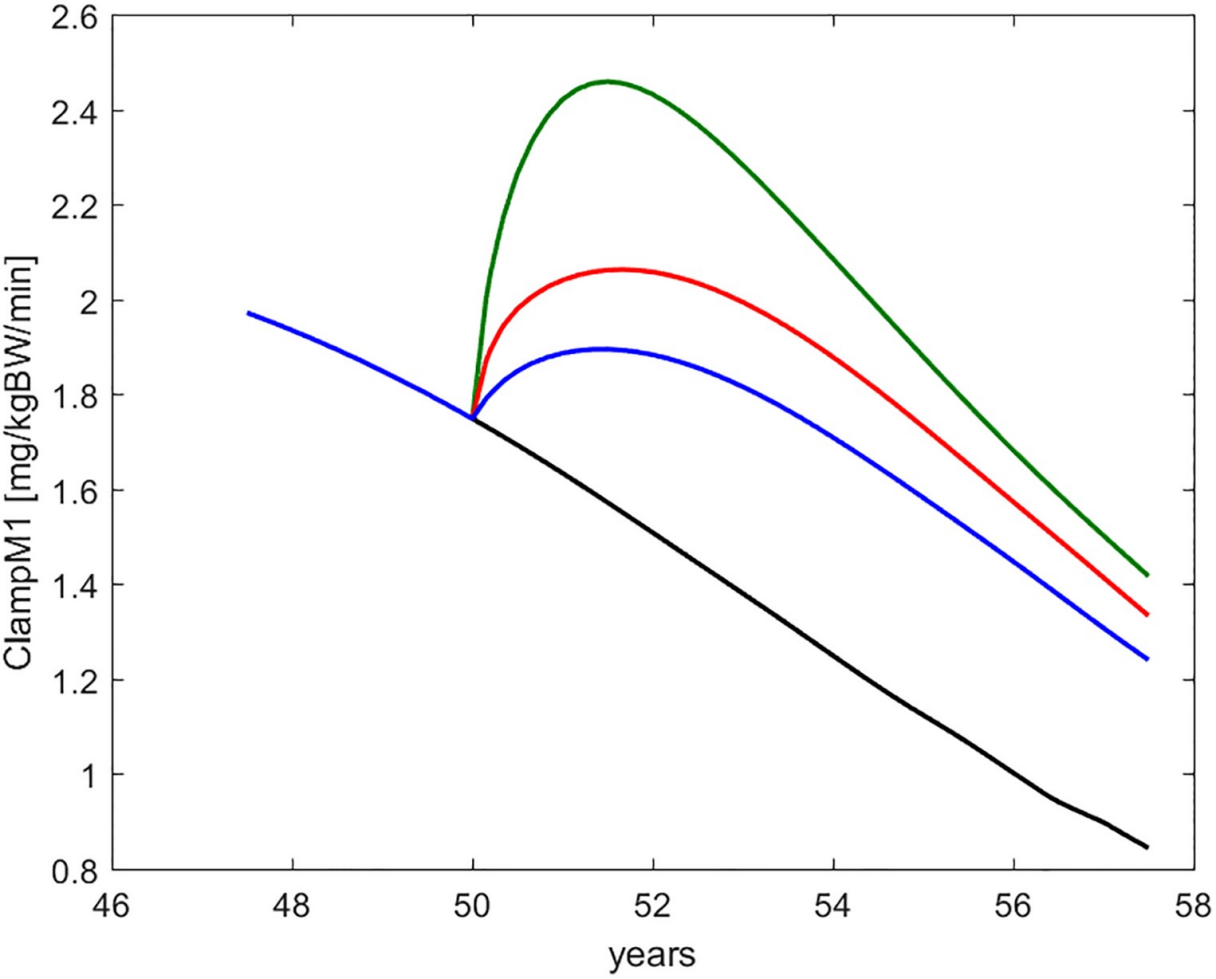
ClampM1. Time course of the first-phase Euglycemic Hyperinsulinemic Clamp M-index, as derived from a simulated clamp study at each time during the life history of the virtual subject. The curves refer to no intervention (black), Placebo (blue), Metformin (red), Intensive LifeStyle (green).

**Fig 12:**
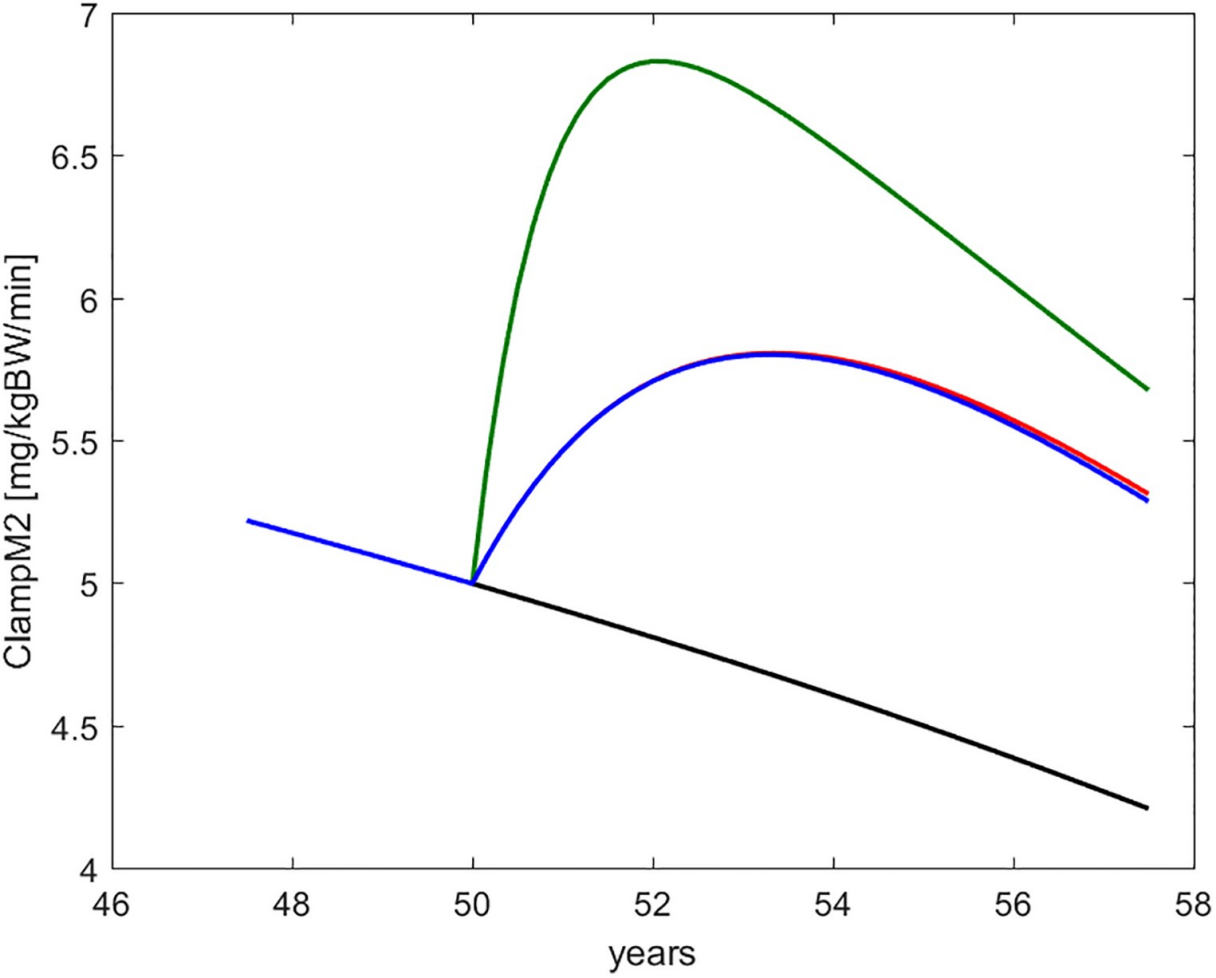
ClampM2. Time course of the second-phase Euglycemic Hyperinsulinemic Clamp M-index, as derived from a simulated clamp study at each time during the life history of the virtual subject. The curves refer to no intervention (black), Placebo (blue), Metformin (red), Intensive LifeStyle (green).

**Fig 13:**
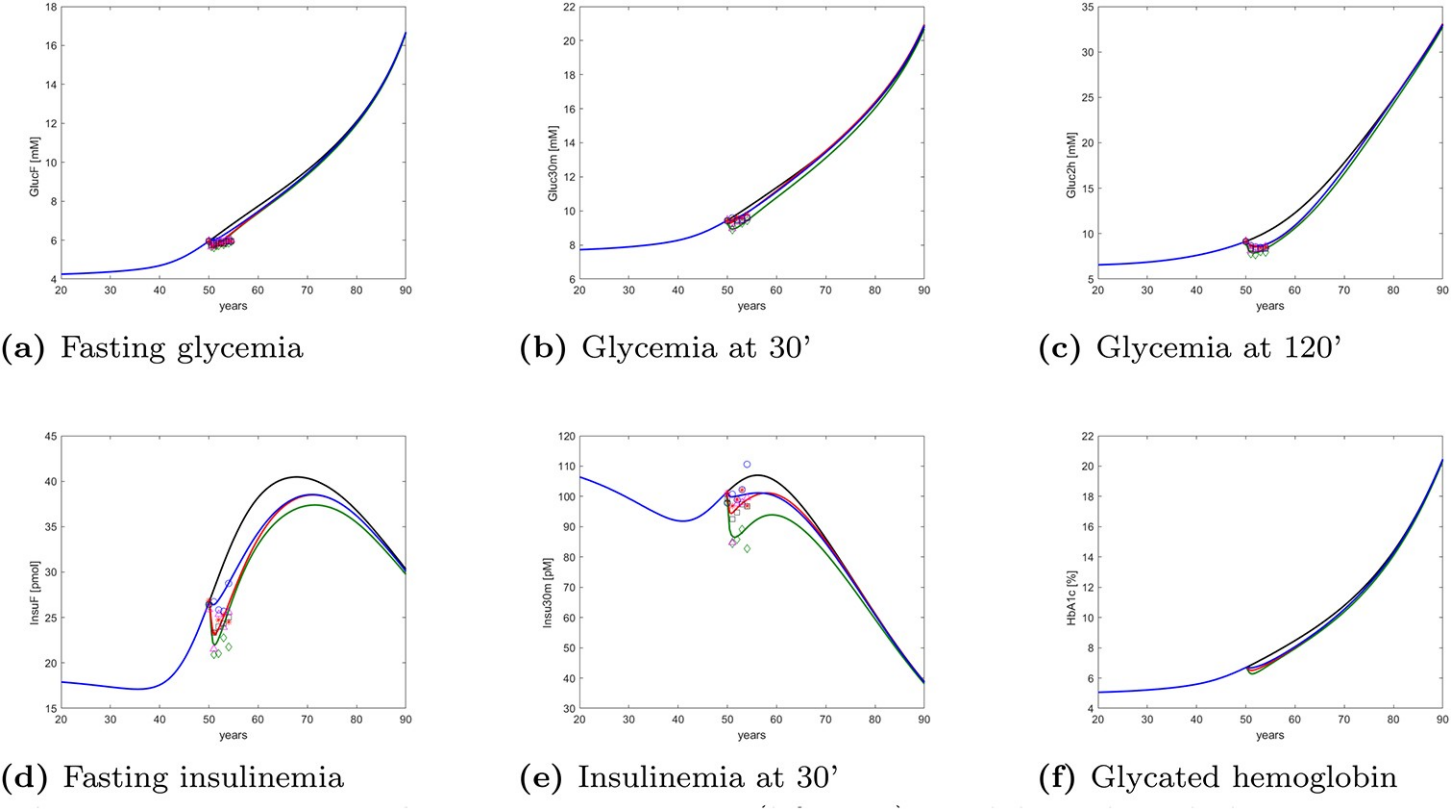
Long-term changes. Long-term (lifetime) model-predicted changes in DPP-measured variables and in HbA1c for the three DPP treatment arms. The curves refer to no intervention (black), Placebo (blue), Metformin (red), Intensive LifeStyle (green). Top panels refer to glycemia (from left to right fasting glycemia, glycemia at 30 minutes during OGTT and glycemia at 2 hours during OGTT). Bottom panels are, from left to right, fasting insulinemia, insulinemia at 30 minutes during OGTT, glycated hemoglobin.

Fig 13 finally shows the model-predicted time course of the five DPP-observed endpoints as well as of glycated hemoglobin, over the whole adult life span of representative virtual subjects, without treatment and undergoing each of the three examined DPP treatment protocols.

We would like to underscore that, of the many variables whose time course is predicted by the model, only five (fasting glycemia, OGTT glycemia at 30 and 120 minutes, fasting insulinemia and OGTT insulinemia at 30 minutes) were actually observed within the DPP study. All of the other variables were not observed (including the complete daily variations of glycemia and insulinemia) and their time courses are inferred by the structure of the model and the fit of the model, with given parameter values, with the actually observed variables.

In the figures, numerical simulations obtained with the DPM15 model are shown together with corresponding sample averages obtained from the Placebo, LifeStyle and Metformin arms of the DPP study. In order to make predictions and average observations comparable, we simulated a representative (“*average*”) subject, reconstructing the subject’s life-trajectory from age 18 (assuming a completely normal insulin sensitivity and secretion profile at this age) until age 50 (approximately the average age of entry into the DPP study [20].

In the last column of Table 2, the values attributed to the parameters under the assumption of no intervention are reported. Table 3 reports those calibrated model parameter values which had to be set differently for the four treatment options considered (no intervention, Placebo, LifeStyle and Metformin). No change in insulin secretory capacity was hypothesized. Improvements in peripheral and hepatic insulin sensitivity are not apparent from the table, as they are determined by relative values of rate of onset, rate of decay and intensity of effect. The maximal improvements are zero for “No treatment”; approximately + 10% and <+ 1% for Placebo; approximately + 22% and + 7% for LifeStyle; approximately + 10% and + 7% for Metformin, all expressed as as percent of normal levels, respectively for peripheral and hepatic insulin sensitivity.

By comparing the values reported in Table 3 with the time-courses of the DPP endpoints in Figs 2 through 6 it can be appreciated how changes in peripheral insulin sensitivity translated mainly into changes in 30 min and 2 hr OGTT glycemia, while changes in hepatic insulin sensitivity translated mainly into changes in fasting glycemia, consistently with common physiological understanding.

The calibration exercise itself provided meaningful information in several ways.

First of all, it was supposed that patient’s glycemias would be progressively increasing, on the average, right before the time of entry into the study: were this not the case, it would be very difficult to imagine that patients could have arrived to pre-diabetic levels from normal levels during young adulthood and that they would then have developed increasing glycemias during the study.

Given the above assumption of increasing glycemias just before enrolment into the study, since even for the Placebo group glycemias were not observed to increase immediately after enrolment (indeed, for the placebo group the fasting glycemia at 6 months was on the average smaller, even if minimally so, than the average fasting glycemia at entry into the study), some clinically significant, even if small, Placebo effect on some of the control variables (peripheral or hepatic insulin sensitivity, gastric emptying rate) must have been present, so we had to conclude that a positive Placebo effect existed.

Secondly, it was not possible to reproduce the observed data assuming the same rapidity of onset and the same rapidity of progressive loss of efficacy of the treatments on each control variable, even assuming possibly different effect sizes. In other words, we could not fit the data assuming that say, metformin and placebo have the same rates of onset and loss of efficacy on peripheral insulin sensitivity, or that metformin and intensive lifestyle differ only in effect size with respect to their impact on hepatic insulin sensitivity. The converse was also true: it was not possible to explain the observations for some treatment, say metformin, by assuming same rapidity of onset and loss of efficacy on different control variables, e.g. on both hepatic and peripheral insulin sensitivity. We had therefore to hypothesize differences in rate of onset and rate of decay of effects for different treatments on the same control variable as well as for the same treatment on different control variables.

The best fits with the observed data for metformin treatment were obtained when we assumed that this intervention acts primarily on hepatic insulin sensitivity. This determined a glucose lowering effect largely limited to fasting glycemia. Interpretation and observation are consistent with studies showing that metformin acts to reduce fasting glucose mainly by decreasing hepatic glucose output and has little effect on peripheral insulin sensitivity [63–65]. Metformin’s specific effect on hepatic insulin sensitivity appeared to be transient; its more modest, but somewhat longer-lasting effects appeared indistinguishable from placebo. The transient nature of metformin’s effects is consistent with previous reports [66–68].

The observed time-course of the recorded variables for the ILS arm was best replicated by the model when assuming an effect on hepatic insulin sensitivity of about the same order of magnitude, and with the same rates of onset and decay, as that hypothesized for Metformin. In addition, however, the observations on the ILS arm were consistent with a very substantial effect on peripheral insulin sensitivity, increasing progressively from the start of treatment, peaking in about two years on average, and, while decreasing thereafter, being substantially maintained over the course of a few more years.

Over the 5-year period of the study the predicted change in *β*-cell mass in a typical subject was greater than 10%, the model predicting in fact the fastest decrease in *β*-cell mass right at this period in the lifetime of the kind of pre-diabetic subjects enrolled in the DPP study. The treatments studied did not appreciably influence, over this time bracket, this apparent rate of loss: model simulations suggest that earlier, more aggressive treatment aimed at drastically reducing insulin resistance and possible additional causes of *β*-cell mass decrement, such as systemic inflammation, would be beneficial.

Finally, while it may be imagined that model parameters could easily be varied so as to arbitrarily change model output, the present model’s structure did not actually allow a wide variety of possible forecasts. Model predictions for the five DPP endpoints are tightly connected: the physiologic assumptions incorporated in the model equations and the relatively small number of free parameters (when compared with the large number of different observations) coerce the predicted curves into rather rigid patterns. Still, parameter values had to be found such that all five DPP endpoints were simultaneously matched when attributing to the different treatments plausible effect sizes and temporal evolutions. This turned out to be in fact possible. In other words, the model incorporates current knowledge in a coherent mathematical structure that cannot be bent to reproduce whatever arbitrarily specified behavior. Such mathematical structure, however, does translate physiologically plausible parameter value changes into realistic predicted time-courses, matching the observed averages in the DPP patient sample.

## Discussion

### Reasons for modelling diabetes progression

It is problematic to test alternative T2DM therapies over the long time-scale of the disease. What data sets exist cover at most a few years for each subject. Mathematical models of disease progression allow meaningful quantitative extrapolation beyond commonly observed time intervals: such models can be used to predict outcomes for novel therapies with anticipated disease-modifying properties and to help guide the design of clinical trials. Simulations can be used to guide sample size and treatment duration based on hypotheses about mechanisms of action (e.g. on insulin resistance or *β*-cell replication), about the magnitude of the effects, about the variability in both disease severity and treatment effects.

The validity of the predictions depends crucially on the robustness of model assumptions and on the mechanistic structure that results from them. The forecasts obtained are the direct expression of what diabetological knowledge is incorporated in the equations. We describe here a model, which we believe is robust and which has enough complexity to provide multiple different outputs allowing the study of multiple, diverse mechanisms of intervention.

### Changes with respect to the previous model

The present work describes a radically improved version of our previous model [10] for the long-term development of Type 2 Diabetes Mellitus and its validation against observations on three patient groups from the Diabetes Prevention Program study [20, 21].

The consideration of a new model was prompted by the need to forecast multiple realistic, testable endpoints (fasting and post-prandial glycemia and insulinemia) rather than restraining oneself to discussing fasting,“representative” or “prevailing” glycemia and insulinemia. This need was met by abandoning the quasi-steady state assumption and by introducing hepatic as well as peripheral insulin sensitivity.

In the previous version of the DPM [10] a classical mathematical approach (singularly perturbed ordinary differential equations by means of small *ϵ*-parameter [69–71]) was employed to harmonize the slow progression of the disease (portrayed by *β*-cell mass or insulin sensitivity) with fast glycemia compensation mechanisms. The classical method considers that, over the few hours of fast dynamics, slow variables are essentially constant, and that, seen from the perspective of decades, whatever fast dynamics occur within a given day can be considered as having converged to equilibrium. Transients are however important in their own right, and, due to meals, the subject is never at equilibrium: the necessary mathematical paradigm shift consisted in discarding the near-equilibrium approach and computing instead, at each slow time step, a complete fast dynamics. This approach not only allows us to separately assess quantities (like glycemia at given times of the day or relative to given meal perturbations), which were lumped together with the previous approach; it also offers us the opportunity of simulating diverse perturbation maneuvers at each slow time and follow over the years some of the most commonly used clinical indices of disease.

It should be noticed that “hepatic insulin sensitivity”, as is commonly understood, refers to the effects of insulin on both liver glucose production and liver glucose uptake. In the context of the present discussion, however, this term only refers to insulin-mediated suppression of Hepatic Glucose Output (HGO): the action of insulin in promoting Peripheral Glucose Disposal (PGD) has been termed “peripheral insulin sensitivity” and refers to muscle, adipose tissue and the liver itself. The second major change with respect to the previous version of the model was then the explicit separation of the action of insulin in suppressing HGO from the action of insulin in promoting PGD.

These two major changes are logically related to each other: in order to express the effects of therapeutic regimens, potentially affecting to different degrees the action of insulin on different target tissues, we need the separate representation of hepatic and peripheral insulin sensitivities, as well as the computation of both fasting levels and post-prandial profiles. Daily oscillations of glycemia and insulinemia were felt to carry important information, which is lost when neglecting transient behavior and considering only equilibrium values. Other recent literature points in the same direction [72].

### Aging and body changes

Once the physiologic part of the model was finalized, we coupled it with equations that describe naturally varying insulin sensitivity and secretion, as well as the time course of the effect of some typical treatment regimens. Peripheral and hepatic insulin sensitivity, as well as functional insulin secretion (maximal insulin secretory ability per Million *β*-cells) are here assumed to decrease gradually over time, with rates depending on genetic and lifestyle factors [41, 73]. At the same time, insulin clearance may slightly decrease in the elderly [41], and the natural restoration or healing capacity of the *β*-cell population (which opposes toxicity to the *β*-cells from whatever cause) may also decline with age [38, 40]. Toxicity to *β*-cells is represented in the current model as depending on (hyper-)glycemic values: while toxicity may well be attributed to lipid products [74], we simplified model structure using glycemia as an indicator of possibly several causes of metabolic *β*-cell impairment.

### Therapy mechanisms

In the present work, all DPP treatments were assumed to affect hepatic and/or peripheral insulin sensitivity (λ_*GI*_ or *k*_*XGI*_, respectively). No treatment effect was hypothesized on insulin secretion ability (*k*_*IB*_) for any of the treatment arms: in fact, it might be postulated that no such direct effect would have been attributed to any of these treatments, unless it was secondary and due to improvement in glucose toxicity.

An initial assumption of identical rates of onset and rates of decay for all effects caused by a given therapy could not be sustained: simulated results based on this assumption could not match the observed data. Conversely, good fits could be obtained by assuming different dynamics for the several effects, which is plausible based on biological and pharmacodynamical principles. In particular, the action of metformin on hepatic insulin sensitivity appeared to be fast and relatively quickly disappearing (whether due to diminished compliance or actual pharmacological loss of effect); instead, metformin appeared to have a slowly appearing and longer lasting action on peripheral insulin sensitivity (in this case indistinguishable from Placebo). This behavior would be plausible if the pharmacologic action of Metformin only affected HGO, while its effect on PGD were due to generic lifestyle changes due to entry into the study).

Conversely, it proved to be reasonable to assume similar rates of onset and decay of effect for both placebo and for the more substantial intensive lifestyle changes: there may be some accumulation of effect over several months as the lifestyle modifications (great or small) produce results, but the positive changes eventually tend to wane as subjects become less compliant.

Some specific conclusions can be drawn by observing what effects should be postulated for each treatment option in order for the corresponding predicted curves to simultaneously approximate all endpoints observed in the DPP study.

The magnitude and time course of the effect on insulin sensitivity produced by the Intensive LifeStyle (ILS) intervention (magnitude that it was necessary to assume in order to adapt model predictions to the observed data) is consistent with the literature: an approximately 20% increase in peripheral insulin sensitivity with ILS is in the range of what has been observed with similar interventions [75–77]. We would expect both hepatic and peripheral insulin sensitivity to improve with weight loss or exercise, with peripheral insulin sensitivity being especially sensitive to exercise and perhaps hepatic sensitivity being at least as responsive to diet. Van der Heijden *et al*. [78] reported 59% and 23% improvement in peripheral and hepatic insulin sensitivity, respectively, in obese adolescents after initiating exercise. Winnick *et al*. [79] assessed changes in peripheral and hepatic insulin sensitivity with exercise: they reported a 63% increase in peripheral insulin sensitivity and no significant change in hepatic insulin sensitivity. Malin *et al*. [80] support the same findings as Winnick. We further note that Ross *et al*. [75], in a group with diet-induced weight loss of approximately 8% (similar to peak mean weight loss in the DPP ILS group), report a 43% increase in clamp M value. The exercise-induced weight loss group in the same study had a 64% increase in M (with approximately 6% weight loss). Malin *et al*. [80] also reported a 53% increase in M after an exercise regimen (without weight loss). Interestingly, exercise did not impact hepatic insulin sensitivity in the Malin study (even though this study may have been underpowered because small). Based on this information from the literature, the increase in peripheral insulin sensitivity, which our model would predict as necessary to reproduce the DPP ILS observations, seems to be very realistic.

The calibrated effects of the considered treatments could reproduce simultaneously the time course of the several observed DPP variables (Figs 2 through 6). From these and the corresponding time courses of common derived clinical indices (Figs 8 through 12) a few interesting considerations emerge. First of all, the effect of metformin on HOMA-IR, HOMA-B and insulinogenic index appears very similar to that of Intensive LifeStyle: this is not surprising since these three indices all reflect either only fasting, or fasting and (rather variable) 30 min glycemias and insulinemias. The role of peripheral insulin sensitivity in attenuating post-prandial glycemic excursions is not fully captured by these indices and is evident only when considering 2 hr OGTT values. Another interesting observation concerns the fact that the model predicts a substantial improvement due to metformin (over and above placebo effect) only in low-insulinization, but not in high-insulinization EHC results: this agrees with the interpretation that the low-insulinization clamp does reflect HGO variability / hepatic insulin sensitivity, while at high serum insulin concentrations HGO is effectively suppressed in any case, and the high-insulinization clamp only reflects the current state of peripheral insulin sensitivity.

The trend of all modelled variables points to a continuing progression of the disease. This is plausible and could be explained by any combination of processes: continued loss of *β*-cells; waning compliance (after initial losses, weight increased over time in the Lifestyle group and medication compliance decreased in both placebo and metformin DPP groups); possible loss of pharmacologic efficacy of metformin over time (consistent with reports by Kahn [66], Brown *et al*. [68], or Ekstrom *et al*. [81]).

The predicted rate of change of the *β*-cell population is relatively small. A thorough analysis of the likely values *in-vivo* of replication and apoptosis rates, hence of the rapidity of *β*-cell mass variation, was conducted previously [10]. Given the assessed parameters, the model predicts both a relatively slow increase in *β*-cell mass following hyperglycemic needs and a relatively slow decline of *β*-cell mass due to toxicity. While otherwise plausible, this behavior might seem inconsistent with the very rapid expansion of *β*-cell mass in pregnancy [82], and with its fast decline after delivery: different mechanisms than those here considered may be at play during pregnancy.

We note that the current model can be used to represent the effects of different therapeutic approaches: not only oral hypoglycemic agents of different kinds (metformin, sulfonylureas, SGLT2 inhibitors, thiazolidinediones) and insulin administration, but also bariatric surgery [83–87] or *β*-cell anti-inflammatory protectors such as Interleukin-1-receptor antagonists [15].

### Parameter calibration

Model parameters were calibrated in order for the forecasts to adapt to DPP data: no statistical parameter estimation was conducted. In the present work the goal was not to fit as closely as possible endpoint averages from a given patient sample, obtaining statistical identification of the model for that specific population, but rather to show how reasonable assumptions on mechanisms of action translate into plausible effect parameters, which in turn translate into realistic matches to observed average behavior. While it can be readily seen that the model structure is consistent with available observations (because there exist parameter values that allow the model to capture observed behavior), no diagnostics of goodness-of-fit are therefore calculated.

It is theoretically possible that apparent physiologic insights from the modeling could be highly specific to the current calibration rather than represent generalizable outcomes. It cannot be claimed that the mechanisms underlying the different responses of DPP subjects to the interventions have been identified. What can be said is that the model structure and the chosen parameter values, consistent with reasonable hypotheses on the underlying physiology, produce outputs with are consistent with the actual observations.

It should be appreciated that, in spite of the large number of free parameters, the tight relationship among the state variables, produced by the mechanistic structure of the model, makes it impossible to obtain whatever behavior is desired for each one of several variables simultaneously, particularly when constraining parameters to physiologically acceptable ranges. Indeed, the very fact that the model is actually able to reproduce observed average behavior of several variables simultaneously on the basis of plausible parameter values is indicative of the robustness of the hypotheses made.

It should also be appreciated that some observations have intrinsically large variability: for example, different patients may have widely varying values of insulinemia at 30’ during OGTT since both the absolute peak values as well as the timing of the insulin peaks vary, and the variability of both is reflected in the large variability of the single observation recorded at 30 minutes. It would be pointless to require that the model agrees with observations more closely than the observations agree among themselves.

### Model features

The model here proposed is rather complex, and choices had to be made for a reasonably parsimonious representation of the many mechanisms involved. These choices attempted to balance adherence to known physiology and mathematical simplicity. For example, gastrointestinal absorption of glucose was represented with a two-compartment, first-order deterministic process (Eqs 17, 18 and 19). Gastric emptying is in fact irregularly intermittent and pulsatile rather than linear and continuous [88]. The traditional simpler single linear elimination (see the exhaustive discussion in Yokrattanasak *et al*. [88]) was deemed sufficient and was adopted here as it speeds up simulations considerably. A constant rate of gastric emptying, such as is posited in Eq 16, represents only a crude first approximation to the likely dynamics of the entry of glucose-rich nutrients into the absorbing bowel. As early as Lehmann and Deutsch [89], the rate of gastric emptying was described as trapezoidal for sufficiently large (> 10 g) carbohydrate intake, with ascending, plateau and descending emptying rate phases. More recently, Li *et al*. [90] investigated functional forms similar respectively to a lognormal distribution or to the right-half of a normal distribution to describe the rate of appearance of glucose (or FFAs) from a mixed meal. On the other hand, Goel *et al*. [91] suggested that while the linear approach used in Eq 16 is appropriate for liquid meals, it may also be used for mixed meals, albeit with a different (slower) dynamics compared to liquid meals. This issue is worth addressing here because, in general, a poor description of the rate of glucose appearance may confound the effects of other glucose transport, secretion or production mechanisms. In the present case, however, precise identification of the glucose absorption dynamics was not the objective, and a simple, qualitatively plausible dynamics manages to produce intra-day or post-OGTT glycemic variations consistent with the observed trends of 30’ and 120’ post-prandial glycemias over several years. In particular, the simple gastric emptying model should be adequate to simulate delivery of nutrients to the small intestine after the OGTT (small volume, liquid, free of fat and protein), hence to reproduce the indices (post-OGTT glycemias at 30’ and 120’, insulinemia at 30’) tracked over slow time by the overall model.

It is of methodological interest to underscore that our model does not prescribe any explicit set-point, either of glycemia (such as is contemplated for instance in the so-called “minimal model” for the IVGTT) or in the fasting glycemia that beta-cells population dynamics may be assumed to target (as in Ribbing *et al*. [11]). Instead, what equilibria exist in the system are determined by the free interplay of the dynamics of the different determinants involved. The apparent set-points are mere emergent features of the complex underlying dynamics, and are apt to change, possibly dramatically so, when this dynamics evolves. The parameter values of the model’s underlying dynamics determine the actual observed values of the apparent set-points, such as the equilibrium level of fasting glycemia in the healthy young adult, as well as the values of other observed features, such as the age of development of diabetes in relation with the degree of insulin resistance.

The interplay between progressively developing insulin resistance and eventually failing compensatory pancreatic insulin hypersecretion is widely considered the hallmark of T2DM, but there are different interpretations (possibly corresponding to actual differences in pathophysiologic mechanisms between patient sub-populations) as to the causal chain leading to the eventual decompensation. It has been hypothesized [92] that some acute event (such as a surgical procedure, or a severe infectious episode), determining a sudden increase in insulin resistance, is responsible for the shift from compensation to decompensation, and some mathematical models of the development of T2DM indeed incorporate such an explicit shift [11, 13, 92]. However, over the clinical course of most T2DM patients it is not possible to identify such a triggering event. The present model makes no recourse to external triggering events and does not need the introduction of an explicit regime shift (a sudden change in parameter values, the introduction of a new external forcing function) in order to reproduce a rapid worsening of the clinical conditions at some point in the life of the subject. Instead, our model formalizes the concept that a persistent hyperglycemic insult, determined by long-standing, possibly progressive degrees of insulin resistance, brings about a progressive decline of insulin sensitivity, and that the even mild glucose toxicity connected with persistent insulin resistance eventually damages pancreatic replication reserve, determines an eventual decline of *β*-cell mass and an eventual failure of compensating insulin hypersecretion, resulting finally in rapid acceleration of hyperglycemia and in the overt clinical picture of frank T2DM.

The DPM15 model does not appear to support the hypothesis that primary insulin hypersecretion might be the causal factor of the development of T2DM. This hypothesis was discussed by Corkey [93] in hypothetical pathophysiological terms and by Goel [94] in mathematical terms, through an adaptation of the original Topp model to include a direct effect of insulin on *β*-cell dynamics. Simulations (not shown) have been conducted with our model assuming no primary insulin resistance (neither peripheral nor hepatic) and assuming conversely that the long-time behavior of glucose-driven insulin secretion by *β*-cells, instead of mildly decreasing with age, actually doubles. In this way the model expresses a progressive insulin hypersecretion, given the same prevailing glucose concentrations. All such simulations fail to reproduce a progressively increasing glycemia up to diabetic levels. The present model has not been modified to include a direct effect of insulin on *β*-cells, also because we agree with the statement [94] that the required causal signal linking insulin resistance and insulin hypersecretion may well be glycemia itself. In the present model the apparent *Corkey paradox* is replicated (glycemia not immediately rising upon worsening of insulin resistance), due to the controlling effects of increased insulin secretion up to the point where relative endocrine pancreatic insufficiency develops. Insulin hypersecretion could in fact be the mechanism by which glucose toxicity exerts its detrimental effects on *β*-cell turnover. Under this hypothesis, while hyperinsulinemia would not by itself be directly toxic, the glucose-induced, prolonged hypersecretory state would be damaging to the *β*-cell. This interpretation is consistent with the role of ‘ER stress’ that has been proposed for *β*-cell death: the overly taxed secretory apparatus of the cell results in accumulation of misfolded proteins which, in turn, trigger inflammatory and apoptotic mechanisms. In all these cases, from a modelling point of view we may take hyperglycemia as a fair indicator of the toxic situation for the *β*-cell population.

### Limitations of the current work

One potential limitation is that the current model does not take into account the possible effect of glucose toxicity on insulin secretory function by existing *β*-cells. The effect of sustained, moderate hyperglycemia is here exerted exclusively on *β*-cell replication, hence on the maintenance of *β*-cell mass: should there be reasons to assume that a toxic effect is also exerted on insulin secretory mechanisms, this ought to be incorporated in the model. This is a moot point however, because current literature reports both increased and decreased insulin secretion with acute hyperglycemia [95].

Another limitation consists in not taking into account lipid metabolism, variations in fat mass, body size etc. as measurable indicators of peripheral insulin resistance and as possible contributing factor to low-key, systemic, continuous inflammation adding its toxic effect on *β*-cell replication.

A third limitation of the current model is its simplistic depiction of (mono-exponential) gastric emptying. Even so, the whole aggregated model captures relevant glycemic oscillations throughout the day, but future work will consider the introduction of a stochastic gastric emptying sub-model as well as the use of stochastically variable meal composition and size.

Finally, model validation against independent sets of observations is clearly desirable and will need to be addressed in the future, similarly to what was done for the previous version of the model in comparing its output with the CANOE study results. [18, 96]

## Conclusion

A new mathematical model of the long-term development of Type 2 Diabetes Mellitus is consistent with available literature, is able to reproduce experimentally observed effects of therapeutic interventions on several endpoints, including fasting and post-prandial glycemias and insulinemias, and can simulate the evolution of common clinical and experimental indices in cohorts of virtual patients. Such an elaborate model will need to be further validated: as it stands now, it incorporates plausible physiology, agrees with available observations, and allows the investigator to formulate quantitative, testable questions for relevant patient populations.

## References

[1] Makroglou A, Li J, Kuang YY (2006) Mathematical models and software tools for the glucose-insulin regulatory system and diabetes: An overview. Applied Numerical Mathematics 56: 559–573. 10.1016/j.apnum.2005.04.023

[2] Ajmera I, Swat M, Laibe C, Le Novère N, Chelliah V (2013) The impact of mathematical modeling on the understanding of diabetes and related complications. CPT Pharmacometrics and Systems Pharmacology 2: e54 10.1038/psp.2013.30 23842097 PMC3731829

[3] Palumbo P, Ditlevsen S, Bertuzzi A, De Gaetano A (2013) Mathematical modeling of the glucose–insulin system: A review. Math Biosci 244: 69–81. 10.1016/j.mbs.2013.05.006 23733079

[4] Palumbo P, Pizzichelli G, Panunzi S, Pepe P, De Gaetano A (2014) Model-based control of plasma glycemia: Tests on populations of virtual patients. Math Biosci 257: 2–10. 10.1016/j.mbs.2014.09.003 25223234

[5] De Gaetano A, Gaz C, Palumbo P, Panunzi S (2015) A unifying organ model of pancreatic insulin secretion. PLoS One 10: e0142344 10.1371/journal.pone.0142344 26555895 PMC4640662

[6] Borri A, Panunzi S, De Gaetano A (2016) A glycemia-structured population model. J Math Biol 73(1): 39–62. 10.1007/s00285-015-0935-7 26440781

[7] Topp BG, Promislow K, deVries G, Miura RM, Finegood DT (2000) A model of beta-cell mass, insulin, and glucose kinetics: pathways to diabetes. J Theor Biol 206: 605–619. 10.1006/jtbi.2000.2150 11013117

[8] Bagust A, Evans M, Beale S, Home PD, Perry AS, Stewart M (2006) A model of long-term metabolic progression of type 2 diabetes mellitus for evaluating treatment strategies. PharmacoEconomics 24S1: 5–19. 10.2165/00019053-200624001-00002 16800159

[9] de Winter W, DeJongh J, Post T, Ploeger B, Urquhart R, Moules I, et al (2006) A mechanism-based disease progression model for comparison of long-term effects of pioglitazone, metformin and gliclazide on disease processes underlying type 2 diabetes mellitus. J Pharmacokinet Pharmacodyn 33: 313–343. 10.1007/s10928-006-9008-2 16552630

[10] De Gaetano A, Hardy T, Beck B, Abu-Raddad E, Palumbo P, Bue-Valleskey J, et al (2008) Mathematical models of diabetes progression. Am J Physiol 295: E1462–E1479. 10.1152/ajpendo.90444.2008 18780774

[11] Ribbing J, Hamrén B, Svensson MK, Karlsson MO (2010) A model for glucose, insulin, and beta-cell dynamics in subjects with insulin resistance and patients with type 2 diabetes. J Clin Pharmacol 50: 861–872. 10.1177/0091270009349711 20484615

[12] Boutayeb W, Lamlili MEN, Boutayeb A, Derouich M (2014) Mathematical modelling and simulation of *β*-cell mass, insulin and glucose dynamics: Effect of genetic predisposition to diabetes. J Biomedical Science and Engineering 7: 330–342. 10.4236/jbise.2014.76035

[13] Palmér R, Nyman E, Penney M, Marley A, Walker G, Cedersund G, et al (2014) Effects of il-1*β*–blocking therapies in type 2 diabetes mellitus: A quantitative systems pharmacology modeling approach to explore underlying mechanisms. CPT Pharmacometrics Syst Pharmacol 3: e118 10.1038/psp.2014.16 24918743 PMC4076803

[14] Ha J, Satin LS, Sherman AS (2016) A mathematical model of the pathogenesis, prevention, and reversal of type 2 diabetes. Endocrinology 157(2): 624–635. 10.1210/en.2015-1564 26709417 PMC4733125

[15] Larsen CM, Faulenbach M, Vaag A, Vølund A, Ehses JA, Seifert B, et al (2007) Interleukin-1-receptor antagonist in type 2 diabetes mellitus. N Engl J Med 356: 1517–1526. 10.1056/NEJMoa065213 17429083

[16] Larsen CM, Faulenbach M, Vaag A, Ehses JA, Donath MY, Mandrup-Poulsen T (2009) Sustained effects of interleukin-1 receptor antagonist treatment in type 2 diabetes. Diabetes Care 32: 1663–1668. 10.2337/dc09-0533 19542207 PMC2732140

[17] Sloan-Lancaster J, Abu-Raddad E, Polzer J, Miller JW, Scherer JC, De Gaetano A, et al (2013) Double-blind, randomized study evaluating the glycemic and anti-inflammatory effects of subcutaneous ly2189102, a neutralizing il-1beta antibody, in patients with type 2 diabetes. Diabetes Care 36(8): 2239–46. 10.2337/dc12-1835 23514733 PMC3714510

[18] Hardy TA, Abu-Raddad E, Porksen N, De Gaetano A (2012) Evaluation of a mathematical model of diabetes progression against observations in the diabetes prevention program. Am J Physiol 303: E200–E212. 10.1152/ajpendo.00421.2011 22550065

[19] Kitabchi AE, Temprosa M, Knowler WC, Kahn SE, Fowler SE, Haffner SM, et al (2005) Role of insulin secretion and sensitivity in the evolution of type 2 diabetes in the diabetes prevention program: effects of lifestyle intervention and metformin. Diabetes 54: 2404–2414. 10.2337/diabetes.54.8.2404 16046308 PMC1360738

[20] Knowler WC, Barrett-Connor E, Fowler SE, Hamman RF, Lachin JM, Walker EA, et al (2002) Reduction in the incidence of type 2 diabetes with lifestyle intervention or metformin. N Engl J Med 346: 393–403. 10.1056/NEJMoa012512 11832527 PMC1370926

[21] Knowler WC, Hamman RF, Edelstein SL, Barrett-Connor E, Ehrmann DA, Walker EA, et al (2005) Prevention of type 2 diabetes with troglitazone in the diabetes prevention program. Diabetes 54: 1150–1156. 10.2337/diabetes.54.4.1150 15793255 PMC1351025

[22] Butler AE, Janson J, Bonner-Weir S, Ritzel R, Rizza RA, Butler PC (2003) Beta-cell deficit and increased beta-cell apoptosis in humans with type 2 diabetes. Diabetes 52: 102–110. 12502499 10.2337/diabetes.52.1.102

[23] Sakuraba H, Mizukami H, Yagihashi N, Wada R, Hanyu C, Yagihashi S (2002) Reduced beta-cell mass and expression of oxidative stress-related dna damage in the islet of japanese type ii diabetic patients. Diabetologia 45(1): 85–96. 10.1007/s125-002-8248-z 11845227

[24] Yoon KH, Ko SH, Cho JH, Lee JM, Ahn YB, Song KH, et al (2003) Selective beta-cell loss and alpha-cell expansion in patients with type 2 diabetes mellitus in korea. J Clin Endocrinol Metab 88(5): 2300–2308. 10.1210/jc.2002-020735 12727989

[25] Rahier J, Wallon J, Loozen S, Lefevre A, Gepts W, Haot J (1983) The pancreatic polypeptide cells in the human pancreas: the effects of age and diabetes. J Clin Endocrinol Metab 56(3): 441–444. 10.1210/jcem-56-3-441 6337179

[26] Street CN, Lakey JR, Shapiro AM, Imes S, Rajotte RV, Ryan EA, et al (2004) Islet graft assessment in the edmonton protocol: implications for predicting long-term clinical outcome. Diabetes 53(12): 3107–3114. 10.2337/diabetes.53.12.3107 15561940

[27] Brissova M, Fowler MJ, Nicholson WE, Chu A, Hirshberg B, Harlan DM, et al (2005) Assessment of human pancreatic islet architecture and composition by laser scanning confocal microscopy. J Histochem Cytochem 53(9): 1087–1097. 10.1369/jhc.5C6684.2005 15923354

[28] Hanley SC, Austin E, Assouline-Thomas B, Kapeluto J, Blaichman J, Moosavi M, et al (2010) *β*-cell mass dynamics and islet cell plasticity in human type 2 diabetes. Endocrinology 151(4): 1462–1472. 10.1210/en.2009-1277 20176718

[29] Van Assche FA, Aerts L, De Prins F (1978) A morphological study of the endocrine pancreas in human pregnancy. Br J Obstet Gynaecol 85(11): 818–820. 10.1111/j.1471-0528.1978.tb15835.x 363135

[30] Hugl SR, White MF, Rhodes CJ (1998) Insulin-like growth factor i (igf-i)-stimulated pancreatic beta-cell growth is glucose-dependent. synergistic activation of insulin receptor substrate-mediated signal transduction pathways by glucose and igf-i in ins-1 cells. J Biol Chem 273: 17771–17779. 9651378 10.1074/jbc.273.28.17771

[31] Pechhold K, Koczwara K, Zhu X, Harrison VS, Walker G, Lee J, et al (2009) Blood glucose levels regulate pancreatic beta-cell proliferation during experimentally-induced and spontaneous autoimmune diabetes in mice. PLoS One 4 10.1371/journal.pone.0004827 PMC2654100 19287497

[32] Porat S, Weinberg-Corem N, Tornovsky-Babaey S, Schyr-Ben-Haroush R, Hija A, Stolovich-Rain M, et al (2011) Control of pancreatic beta cell regeneration by glucose metabolism. Cell Metab 13: 440–449. 10.1016/j.cmet.2011.02.012 21459328 PMC11807376

[33] Yki-Jarvinen H (1992) Glucose toxicity. Endocr Rev 13: 415–431. 10.1210/edrv-13-3-415 1425483

[34] Yki-Jarvinen H (1998) Toxicity of hyperglycaemia in type 2 diabetes. Diabetes Metab Rev 14 Suppl 1: S45–S50. 9816487

[35] Donath MY, Halban PA (2004) Decreased beta-cell mass in diabetes: significance, mechanisms and therapeutic implications. Diabetologia 47(3): 581–589. 10.1007/s00125-004-1336-4 14767595

[36] Ritzel RA, Butler AE, Rizza RA, Veldhuis JD, Butler PC (2006) Relationship between beta-cell mass and fasting blood glucose concentration in humans. Diabetes Care 29(3): 717–718. 10.2337/diacare.29.03.06.dc05-1538 16505537

[37] Maedler K, Spinas GA, Lehmann R, Sergeev P, Weber M, Fontana A, et al (2001) Glucose induces *β*-cell apoptosis via upregulation of the Fas receptor in human islets. Diabetes 50: 1683–1690. 10.2337/diabetes.50.8.1683 11473025

[38] Maedler K, Schumann DM, Schulthess F, Oberholzer J, Bosco D, Berney T, et al (2006) Aging correlates with decreased *β*-cell proliferative capacity and enhanced sensitivity to apoptosis. Diabetes 55: 2455–2462. 16936193 10.2337/db05-1586

[39] Reers C, Erbel S, Esposito I, Schmied B, Büchler MW, Nawroth PP, et al (2009) Impaired islet turnover in human donor pancreata with aging. Eur J Endocrinol 160: 185–191. 10.1530/EJE-08-0596 19004984

[40] Tyrberg B, Eizirik DL, Hellerstrom C, Pipeleers DG, Andersson A (1996) Human pancreatic beta-cell deoxyribonucleic acid-synthesis in islet grafts decreases with increasing organ donor age but increases in response to glucose stimulation in vitro. Endocrinology 137: 5694–5699. 8940401 10.1210/endo.137.12.8940401

[41] Iozzo P, Beck-Nielsen H, Laakso M, Smith U, Yki-Jarvinen H, Ferrannini E (1999) Independent influence of age on basal insulin secretion in nondiabetic humans. J Clin Endocrinol Metab 84: 863–868. 10.1210/jcem.84.3.5542 10084562

[42] Wilcox G (2005) Insulin and insulin resistance. Clin Biochem Rev 26(2): 19–39. 16278749 PMC1204764

[43] Cherrington AD (1999) Banting lecture 1997. control of glucose uptake and release by the liver in vivo. Diabetes 48(5): 1198–1214. 10.2337/diabetes.48.5.1198 10331429

[44] Iozzo P, Geisler F, Oikonen V, Mäki M, Takala T, Solin O, et al (2003) Insulin stimulates liver glucose uptake in humans: an 18f-fdg pet study. J Nucl Med 44(5): 682–689. 12732668

[45] Basu R, Basu A, Johnson CM, Schwenk WF, Rizza RA (2004) Insulin dose-response curves for stimulation of splanchnic glucose uptake and suppression of endogenous glucose production differ in nondiabetic humans and are abnormal in people with type 2 diabetes. Diabetes 53: 2042–2050. 10.2337/diabetes.53.8.2042 15277384

[46] Nurjhan N, Campbell PJ, Kennedy FP, Miles JM, Gerich JE (1986) Insulin dose-response characteristics for suppression of glycerol release and conversion to glucose in humans. Diabetes 35: 1326–1331. 10.2337/diab.35.12.1326 3533681

[47] Groop LC, Bonadonna RC, DelPrato S, Ratheiser K, Zyck K, Ferrannini E, et al (1989) Glucose and free fatty acid metabolism in non-insulin-dependent diabetes mellitus: Evidence for multiple sites of insulin resistance. J Clin Invest 84: 205–213. 10.1172/JCI114142 2661589 PMC303971

[48] Panunzi S, Palumbo P, De Gaetano A (2007) A discrete single delay model for the intra-venous glucose tolerance test. Theor Biol Med Model 4: 35 10.1186/1742-4682-4-35 17850652 PMC2072949

[49] Palumbo P, Panunzi S, De Gaetano A (2007) Qualitative behavior of a family of delay differential models of the glucose insulin system. Discrete and Continuous Dynamical Systems—Series B 7: 399–424.

[50] Panunzi S, Mingrone G, De Gaetano A (2010) Advantages of the single delay model for the assessment of insulin sensitivity from the intravenous glucose tolerance test. Theor Biol Med Model 7: 9 10.1186/1742-4682-7-9 20298586 PMC2858103

[51] De Gaetano A, Panunzi S, Matone A, Samson A, Vrbikova J, Bendlova B, et al (2013) Routine OGTT: A robust model including incretin effect for precise identification of insulin sensitivity and secretion in a single individual. PLoS One 8: e70875 10.1371/journal.pone.0070875 24009656 PMC3756988

[52] Rizza RA, Mandarino LJ, Gerich JE (1981) Dose-response characteristics for effects of insulin on production and utilization of glucose in man. Am J Physiol 240: E630–E639. 10.1152/ajpendo.1981.240.6.E630 7018254

[53] Alford FP, Bloom SR, Nabarro JD (1976) Glucagon metabolism in man, studies on the metabolic clearance rate and the plasma acute disappearance time of glucagon in normal and diabetic subjects. J Clin Endocrinol Metab 42: 830–838. 10.1210/jcem-42-5-830 773949

[54] Fisher M, Sherwin RS, Hendler R, Felig P (1976) Kinetics of glucagon in man: effects of starvation. Proc Natl Acad Sci USA 73: 1735–1739. 10.1073/pnas.73.5.1735 1064045 PMC430375

[55] Jaspan JB, Polonsky KS, Lewis M, Pensler J, Pugh W, Moossa AR, et al (1981) Hepatic metabolism of glucagon in the dog: contribution of the liver to overall metabolic disposal of glucagon. Am J Physiol 240: E233–E244. 10.1152/ajpendo.1981.240.3.E233 7011049

[56] Konig M, Bulik S, Holzhutter HG (2012) Quantifying the contribution of the liver to glucose homeostasis: a detailed kinetic model of human hepatic glucose metabolism. PLoS Comput Biol 8 10.1371/journal.pcbi.1002577 PMC3383054 22761565

[57] Gerich J, Davis J, Lorenzi M, Rizza R, Bohannon N, Karam J, et al (1979) Hormonal mechanisms of recovery from insulin-induced hypoglycemia in man. Am J Physiol 236: E380–E385. 10.1152/ajpendo.1979.236.4.E380 434200

[58] Cavallo-Perin P, Bruno A, Scaglione L, Gruden G, Cassader M, Pagano G (1993) Feedback inhibition of insulin and glucagon secretion by insulin is altered in abdominal obesity with normal or impaired glucose tolerance. Acta Diabetol 30: 154–158. 10.1007/BF00572860 8111076

[59] Elahi D, Nagulesparan M, Hershcopf RJ, Muller DC, Tobin JD, Blix PM, et al (1982) Feedback inhibition of insulin secretion by insulin: relation to the hyperinsulinemia of obesity. N Engl J Med 306: 1196–1202. 10.1056/NEJM198205203062002 7040963

[60] Cryer PE, Davis SN, Shamoon H (2003) Hypoglycemia in diabetes. Diabetes Care 26: 1902–1912. 10.2337/diacare.26.6.1902 12766131

[61] GGerich JE, Langlois M, Noacco C, Karam JH, Forsham PH (1973) Lack of glucagon response to hypoglycemia in diabetes: evidence for an intrinsic pancreatic alpha cell defect. Science 182: 171–173. 10.1126/science.182.4108.171 4581053

[62] De Gaetano A, Panunzi S, Eliopoulos D, Hardy T, Mingrone G (2014) Mathematical modeling of renal tubular glucose absorption after glucose load. PLoS One 9: e86963 10.1371/journal.pone.0086963 24489817 PMC3906102

[63] DeFronzo RA, Barzilai N, Simonson DC (1991) Mechanism of metformin actionin obese and lean noninsulin-dependent diabetic subjects. J Clin Endocrinol Metab 73: 1294–1301. 10.1210/jcem-73-6-1294 1955512

[64] Stumvoll M, Nurjhan N, Perriello G, Dailey G, Gerich J (1995) Metabolic effects of metformin in non-insulin-dependent diabetes mellitus. N Engl J Med 333: 550–554. 10.1056/NEJM199508313330903 7623903

[65] Inzucchi SE, Maggs DG, Spollett GR, Page SL, Rife FS, Walton V, et al (1998) Efficacy and metabolic effects of metformin and troglitazone in type ii diabetes mellitus. N Engl J Med 338: 867–872. 10.1056/NEJM199803263381303 9516221

[66] Kahn SE, Haffner SM, Heise MA, Herman WH, Holman RR, Jones NP, et al (2006) Glycemic durability of rosiglitazone, metformin, or glyburide monotherapy. N Engl J Med 355:23: 2427–2443. 10.1056/NEJMoa066224 17145742

[67] Nichols GA, Alexander CM, Girman CJ, Kamal-Bahl SJ, Brown JB (2006) Treatment escalation and rise in hba1c following successful initial metformin therapy. Diabetes Care 29: 504–509. 10.2337/diacare.29.03.06.dc05-1937 16505496

[68] Brown JB, Conner C, Nichols GA (2010) Secondary failure of metformin monotherapy in clinical practice. Diabetes Care 33: 501–506. 10.2337/dc09-1749 20040656 PMC2827496

[69] Tikhonov AN (1948) On the dependence of the solutions of differential equations on a small parameter. Matimaticheskii Sbornik (NS) 22 (64).

[70] Artstein Z (2010) Analysis and control of coupled slow and fast systems: a review. Proceedings of the 9th Brazilian 1254–1263.

[71] Zagaris A, Kaper HG, Kaper TJ (2005) Two perspectives on reduction of ordinary differential equations. Math Nachr 278: 1629–1642. 10.1002/mana.200410328

[72] Wang YF, Khan M, van den Berg HA (2012) Interaction of fast and slow dynamics in endocrine control systems with an application to beta-cell dynamics. Math Biosci 235: 8–18. 10.1016/j.mbs.2011.10.003 22063267

[73] Haffner SM, D’Agostino R Jr, Festa A, Bergman RN, Mykkänen L, Karter A, et al (2003) Low insulin sensitivity (s(i) = 0) in diabetic and nondiabetic subjects in the insulin resistance atherosclerosis study: is it associated with components of the metabolic syndrome and nontraditional risk factors? Diabetes Care 26(10): 2796–2803. 10.2337/diacare.26.10.2796 14514582

[74] McGarry JD, Dobbins RL (1999) Fatty acids, lipotoxicity and insulin secretion. Diabetologia 42(2): 128–138. 10.1007/s001250051130 10064091

[75] Ross R, Dagnone D, Jones PJ, Smith H, Paddags A, Hudson R, et al (2000) Reduction in obesity and related comorbid conditions after diet-induced weight loss or exercise-induced weight loss in men. Ann Intern Med 133: 92–103. 10.7326/0003-4819-133-2-200007180-00008 10896648

[76] Houmard JA, Tanner CJ, Slentz CA, Duscha BD, McCartney JS, Kraus WE (2004) Effect of the volume and intensity of exercise training on insulin sensitivity. J Appl Physiol 96: 101–106. 10.1152/japplphysiol.00707.2003 12972442

[77] Mayer-Davis EJ, D’Agostino R Jr, Karter AJ, Haffner SM, Rewers MJ, Saad M, et al (1998) Intensity and amount of physical activity in relation to insulin sensitivity: the insulin resistance atherosclerosis study. JAMA 279: 669–674. 10.1001/jama.279.9.669 9496984

[78] van der Heijden GJ, Toffolo G, Manesso E, Sauer PJ, Sunehag AL (2009) Aerobic exercise increases peripheral and hepatic insulin sensitivity in sedentary adolescents. J Clin Endocrinol Metab 94(11): 4292–4299. 10.1210/jc.2009-1379 19808855 PMC2775656

[79] Winnick JJ, Sherman WM, Habash DL, Stout MB, Failla ML, Belury MA, et al (2008) Short-term aerobic exercise training in obese humans with type 2 diabetes mellitus improves whole-body insulin sensitivity through gains in peripheral, not hepatic insulin sensitivity. J Clin Endocrinol Metab 93: 771–778. 10.1210/jc.2007-1524 18073312 PMC2266960

[80] Malin SK, Gerber R, Chipkin SR, Braun B (2012) Independent and combined effects of exercise training and metformin on insulin sensitivity in individuals with prediabetes. Diabetes Care 35(1): 131–136. 10.2337/dc11-0925 22040838 PMC3241331

[81] Ekström N, Svensson AM, Miftaraj M, Andersson Sundell K, Cederholm J, Zethelius B, et al (2015) Durability of oral hypoglycemic agents in drug naïve patients with type 2 diabetes: report from the swedish national diabetes register (ndr). BMJ Open Diabetes Research and Care 3: e000059 10.1136/bmjdrc-2014-000059 25815205 PMC4368982

[82] Butler AE, Cao-Minh L, Galasso R, Rizza RA, Corradin A, Cobelli C, et al (2010) Adaptive changes in pancreatic beta cell fractional area and beta cell turnover in human pregnancy. Diabetologia 53(10): 2167–2176. 10.1007/s00125-010-1809-6 20523966 PMC2931643

[83] Sjöström L, Peltonen M, Jacobson P, Ahlin S, Andersson-Assarsson J, Anveden Å, et al (2014) Association of bariatric surgery with long-term remission of type 2 diabetes and with microvascular and macrovascular complications. JAMA 311(22): 2297–2304. 10.1001/jama.2014.5988 24915261

[84] Courcoulas AP, Belle SH, Neiberg RH, Pierson SK, Eagleton JK, Kalarchian MA, et al (2015) Three-year outcomes of bariatric surgery vs lifestyle intervention for type 2 diabetes mellitus treatment a randomized clinical trial. JAMA Surg 150(10): 940 10.1001/jamasurg.2015.1534 26132586 PMC4905566

[85] Mingrone G, Panunzi S, De Gaetano A, Guidone C, Iaconelli A, Nanni G, et al (2015) Bariatric-metabolic surgery versus conventional medical treatment in obese patients with type 2 diabetes: 5 year follow-up of an open-label, single-centre, randomised controlled trial. Lancet 386: 964–973. 10.1016/S0140-6736(15)00075-6 26369473

[86] Panunzi S, De Gaetano A, Carnicelli A, Mingrone G (2015) Predictors of remission of diabetes mellitus in severely obese individuals undergoing bariatric surgery: Do BMI or procedure choice matter? a meta-analysis. Ann Surg 261: 459–467. 10.1097/SLA.0000000000000863 25361217

[87] Panunzi S, Carlsson L, De Gaetano A, Peltonen M, Rice T, Sjöström L, et al (2016) Determinants of diabetes remission and glycemic control after bariatric surgery. Diabetes Care 39: 166–174. 10.2337/dc15-0575 26628418

[88] Yokrattanasak J, De Gaetano A, Panunzi S, Satiracoo P, Lawton WM, Lenbury Y (2016) A simple, realistic stochastic model of gastric emptying. PLoS One 11(4): e0153297 10.1371/journal.pone.0153297 27057750 PMC4825969

[89] Lehmann ED, Deutsch T (1992) A physiological model of glucose-insulin interaction in type 1 diabetes mellitus. J Biomed Eng 14: 235–242. 10.1016/0141-5425(92)90058-S 1588781

[90] Li Y, Chow CC, Courville AB, Sumner AE, Periwal V (2016) Modeling glucose and free fatty acid kinetics in glucose and meal tolerance test. Theor Biol Med Model 13:8: 1–20. 26934990 10.1186/s12976-016-0036-3 PMC4776401

[91] Goel P, Parkhi D, Barua A, Shah M, Ghaskadbi S (2008) A minimal model approach for analyzing continuous glucose monitoring in type 2 diabetes. Front Physiol 9:673: 1–8. 10.3389/fphys.2018.00673 PMC5994993 29915545

[92] Mason CC, Hanson RL, Knowler WC (2007) Progression to type 2 diabetes characterized by moderate then rapid glucose increases. Diabetes 56: 2054–2061. 10.2337/db07-0053 17473220

[93] Corkey BE (2012) Diabetes: Have we got it all wrong? Diabetes Care 35: 2432–2437. 23173132 10.2337/dc12-0825 PMC3507569

[94] Goel P (2015) Insulin resistance or hypersecretion? the *β*ig picture revisited. J Theor Biol 384: 131–139. 10.1016/j.jtbi.2015.07.033 26300065

[95] Toschi E, Camastra S, Sironi A, Masoni A, Gastaldelli A, Mari A, et al (2002) Effect of acute hyperglycemia on insulin secretion in humans. Diabetes 51S1: S130–S133. 10.2337/diabetes.51.2007.S130 11815471

[96] Retnakaran R, Qi Y, Harris SB, Hanley AJ, Zinman B (2011) Changes over time in glycemic control, insulin sensitivity, and beta-cell function in response to low-dose metformin and thiazolidinedione combination therapy in patients with impaired glucose tolerance. Diabetes Care 34: 1601–1604. 10.2337/dc11-0046 21709296 PMC3120173

